# Characterization of Japanese Plum (*Prunus salicina*) PsMYB10 Alleles Reveals Structural Variation and Polymorphisms Correlating With Fruit Skin Color

**DOI:** 10.3389/fpls.2021.655267

**Published:** 2021-06-08

**Authors:** Arnau Fiol, Beatriz E. García-Gómez, Federico Jurado-Ruiz, Konstantinos Alexiou, Werner Howad, Maria José Aranzana

**Affiliations:** ^1^Centre for Research in Agricultural Genomics, CSIC-IRTA-UAB-UB, Campus UAB, Barcelona, Spain; ^2^Institut de Recerca i Tecnologia Agroalimentàries, Barcelona, Spain

**Keywords:** MYB10, anthocyanins, fruit color, structural variability, marker-assisted selection, Japanese plum, Asian plum

## Abstract

The red to blue hue of plant organs is caused due to anthocyanins, which are water-soluble flavonoid pigments. The accumulation of these pigments is regulated by a complex of R2R3-MYB transcription factors (TFs), basic-helix-loop-helix (bHLH), and WD-repeat (WDR) proteins (MBW complex). In Rosaceae species, R2R3-MYBs, particularly MYB10 genes, are responsible for part of the natural variation in anthocyanin colors. Japanese plum cultivars, which are hybrids of *Prunus salicina*, have high variability in the color hue and pattern, going from yellow-green to red and purple-blue, probably as a result of the interspecific hybridization origin of the crop. Because of such variability, Japanese plum can be considered as an excellent model to study the color determination in Rosaceae fruit tree species. Here, we cloned and characterized the alleles of the *PsMYB10* genes in the linkage group LG3 region where quantitative trait loci (QTLs) for the organ color have been mapped to other *Prunus* species. Allele segregation in biparental populations as well as in a panel of varieties, combined with the whole-genome sequence of two varieties with contrasting fruit color, allowed the organization of the MYB10 alleles into haplotypes. With the help of this strategy, alleles were assigned to genes and at least three copies of *PsMYB10.1* were identified in some varieties. In total, we observed six haplotypes, which were able to characterize 91.36% of the cultivars. In addition, two alleles of *PsMYB10.1* were found to be highly associated with anthocyanin and anthocyanin-less skin. Their expression during the fruit development confirms their role in the fruit skin coloration. Here, we provide a highly efficient molecular marker for the early selection of colored or non-colored fruits in Japanese plum breeding programs.

## Introduction

Anthocyanins are water-soluble flavonoid pigments that confer the purple-blue hue to plant organs. In flowers and fruits, these pigments enhance flower pollination and seed dispersion mediated by animals; in vegetative tissues, they provide tolerance to abiotic stresses including photoprotection after exposure to long-term light stress, resistance to chilling and desiccation, and recovery from mechanical injury (Winkel-Shirley, 2002; Kong et al., 2003; Gould, 2004). The antioxidant activity of anthocyanins has been further linked to human health-promoting effects when incorporated in the diet. The list of beneficial effects includes anticarcinogenic and anti-inflammatory activity and the prevention of cardiovascular diseases, diabetes, and obesity (Khoo et al., 2017).

Anthocyanins are synthesized through a complex regulatory mechanism for the final appropriate pigment levels to meet the demands of plant development and environmental responses (Albert et al., 2014a,b). In eudicots, biosynthetic genes of anthocyanins are mostly transcriptionally activated by a complex consisting of R2R3-MYB transcription factors (TFs), basic-helix-loop-helix (bHLH), and WD-repeat (WDR) proteins (MBW complex), that induces the expression of several genes in the anthocyanin biosynthesis pathway by binding directly to their promoters (Koes et al., 2005; Gonzalez et al., 2008). Allelic variants of R2R3-MYB genes, including changes in the promoter and coding regions, or even a variation of methylation levels in the promoter and gene bodies, may produce significant changes in the biosynthesis of anthocyanins, causing a variation in fruit coloring (Espley et al., 2007; Telias et al., 2011; Wang et al., 2013; Lü et al., 2018) indicating that R2R3-MYBs are the keys to specify the action of the MBW complex. R2R3-MYB TFs are activated by developmental or environmental signals (Feng et al., 2013; Vimolmangkang et al., 2014; An et al., 2020a). Likewise, the TFs involved in light and hormone signaling pathways may regulate the biosynthesis of anthocyanins directly or indirectly (Shin et al., 2013; Wang et al., 2018; Zhang et al., 2018; An et al., 2020b; Li et al., 2020). MYB TFs can also act as a negative regulator of anthocyanin levels by repressing the expression of anthocyanin genes, possibly by recruiting MBW complexes (Albert et al., 2009; Zhou et al., 2019) or by retaining the ability to form an MBW complex and target DNA motifs when the MYB alleles are truncated (Paz-Ares et al., 1990; Gonzalez et al., 2008; Velten et al., 2010).

Rosaceous fruits such as apples, pears, peaches, apricots, plums, cherries, and strawberries are broadly considered as a source of anthocyanins. In these fruits, such pigments are present in the skin and the flesh, overlying chlorophylls and carotenoids from the fruit development till ripening. External factors such as sunlight, cold temperatures, and crop management are the keys in color development and patterning. Fruit color has an important impact on the choice of consumer, but, while the consumers are interested in this and other traditional quality traits related to flavor, they also demand attributes including nutritional quality. Skin fruit color is a readily observable trait for variety identification. In some fruits, red color is associated with ripeness and better taste and flavor, and red fruits are also highly valued for their content of healthy compounds. Therefore, there is considerable interest in breeding these crops to obtain new varieties bearing fruits with diverse colors, hues, and patterns, while enhancing fruit nutritional quality (Ogah et al., 2014; Panche et al., 2016; García-Gómez et al., 2021). Such breeding efforts have been accompanied by a growing scientific interest in understanding the molecular mechanisms underlying the biosynthesis and accumulation of anthocyanins.

The developmental regulatory network and specific regulators of the biosynthesis of anthocyanins have been studied in most of the major rosaceous fruit species. Structural genes involved in the early and late anthocyanin biosynthetic path have been isolated (Honda et al., 2002; Ubi et al., 2006; Fischer et al., 2007; García-Gómez et al., 2020) and the MYB genes responsible for the observed variation in the color and pattern in plant organs. Apple (*Malus x domestica*) is a rosaceous crop where the anthocyanin regulation has been more studied, where three MYB genes (*MdMYB1, MdMYBA*, and *MdMYB10*) controlling the biosynthesis of anthocyanins in the skin and/or the fruit cortex have been isolated and their function is validated (Takos et al., 2006; Ban et al., 2007; Espley et al., 2007). Their high amino acid sequence identity and same mapping position in the linkage group LG9 (Chagné et al., 2007; Kumar and Pandey, 2013) suggest that all three are alleles of a single gene (Lin-Wang et al., 2010; Telias et al., 2011). Two additional genes, *MdMYB110a* [paralog of *MdMYB10* (Chagné et al., 2013) and *MdMYB114* (Jiang et al., 2019b)], have been found to be highly expressed in the cortex and the fruit skin, respectively, in correlation with anthocyanin levels. The genomic organization of flavonoid genes is comparable between *Malus* and its related genera *Pyrus*, which has allowed the elucidation of the main flavonoid pathway in *Pyrus* using a homology-based cloning approach (Fischer et al., 2007). In the transcriptional level, *Pyrus sp. PyMYB10, PyMYB114, Pyrus comunis PcMYB10*, and *Pyrus bretschneideri PbMYB10b* and *PbMYB9* act as activators of the anthocyanin pathway (Feng et al., 2010; Zhai et al., 2016; Yao et al., 2017; Zhang et al., 2020). Several studies on both apple and pear sport mutants differing in fruit skin color intensity or pattern have identified hypermethylated regions in the promoter or gene bodies of some of these MYB TFss, most likely preventing their expression, cause a variation in fruit color (Telias et al., 2011; Xu et al., 2012; Wang et al., 2013; El-Sharkawy et al., 2015).

In apples and pears, the *MYB10* mapping region in LG9 is collinear with a region in *Prunus* LG3 (Illa et al., 2011) where some traits related to the plant organ color have been mapped (Espley et al., 2007; Sooriyapathirana et al., 2010; Socquet-Juglard et al., 2013; Donoso et al., 2016). This region contains a cluster of *MYB10* genes, although their number differs between the *Prunus* species. In a peach (*Prunus persica*) and an almond (*Prunus dulcis*), there are three *MYB10* genes (*PpMYB10.1, PpMYB10.2*, and *PpMYB10.3*) in this cluster (Verde et al., 2017; Alioto et al., 2020), while this number rises to five annotated MYBs in the syntenic region of a sweet cherry (*Prunus avium*) (Shirasawa et al., 2017). In these species, MYB10 TFs have been described as positive regulators of the biosynthesis of anthocyanins, and allelic variants have been found to be highly correlated with a variation in fruit color (Tuan et al., 2015; Jin et al., 2016; Bretó et al., 2017; Guo et al., 2020). Similarly, Wang et al. (2020) identified an *MYB10* colorless specific allele in strawberry, a rosaceous crop with a different fruit type, indicating that this mechanism is conserved within the Rosaceae family.

Within the rosaceous crops, Japanese plum is among those with the highest fruit color variation, in both tonality and pattern, especially in the skin, where it can range from anthocyanin-less green and yellow to red, purple, or blue hues. In addition, the skin pigmentation does not necessarily fully cover the fruit, forming patterns with the visible fruit flesh color as a background. Molecular markers suitable for marker-assisted selection (MAS) would be of great value for breeders in order to predict at the seedling stage the color of the fruit that will be produced in 3–4 years. Despite several efforts to map and identify the markers associated with the Japanese plum fruit color, reliable markers for their use in breeding programs are still lacking. González et al. (2016c) designed three EST-SSR markers targeting *PsMYB10.1, PsMYB1*, and *PsbHLH35* to determine the genetic structure of 29 Japanese plum cultivars with different skin colorations, finding that all the yellow-skinned cultivars grouped in a cluster together with some red-skinned cultivars. Later, Salazar et al. (2017) applied GBS for the quantitative trait locus (QTL) mapping of several fruit quality traits in a Japanese plum F1 population, mostly with red or purple skin. The red/purple skin color traits were mapped to LG3 and LG4. One single-nucleotide polymorphism (SNP), close to the *PsMYB10.1* gene, largely explained the tendency for purple fruit skin. All together suggest the role of the LG3 MYB10 region in the determination of the fruit color in Japanese plum.

Japanese plum is a species with a diploid genome derived from the hybridization of *Prunus salicina* with several other species of the Prunophora subgenus, which increases the complexity of the analysis of the genes responsible for color variation in the absence of a reference genome. To date, databases include a number of MYB10 read sequences obtained from Rosaceae crops, including Japanese plum. Their alignment and phylogenetic analysis have shed some light on MYB variability within the family (Lin-Wang et al., 2010); however, a high homology between the MYB factors, their genome localization in a cluster, and high variability especially in non-coding regions hamper its separation into genes or alleles of the same gene.

The main objective of this study is to analyze the genetic variability of the LG3 MYB10 genes in the Japanese plum germplasm and to find possible correlations with fruit anthocyanin color. Here, we have identified and assigned the allelic variants to different *MYB10* genes by means of whole-genome sequencing, *MYB10* allele cloning, phylogeny, and phasing through progeny analysis. We have found high genetic variability in the Japanese plum LG3 *MYB10* gene cluster, which contains three copies of the *MYB10.1* gene in at least some Japanese cultivars. Through the allele and haplotype association analysis, we have identified haplotypes that can predict the fruit skin color with high efficiency in seedlings. These haplotypes can be easily obtained with a single PCR reaction, being highly useful for MAS. Possible reasons and implications of different copies of MYB10.1 genes and allele combinations are discussed.

## Materials and Methods

### Plant Material, Phenotyping, and Nucleic Acid Extraction

The plant material analyzed was from accessions and progenies. The accessions were 81 Japanese plum advanced breeding lines (ABL) from a commercial breeding program and 31 commercial cultivars. The progenies were six F1 biparental families obtained after crossing 10 of the advanced breeding cultivars: C9 × C6 (83 individuals, 49 with fruits phenotyped; from now on referred to as P1), C19 × C26 (111 individuals, 79 of them phenotyped; P2), C14 × C4 (48 individuals, 37 with fruits phenotyped; P3), C11 × C8 (43 individuals, 30 with fruits phenotyped; P4), C8 × C5 (64 individuals, 10 with fruits phenotyped; P5), and C11 × C66 (33 individuals, 14 with fruits phenotyped; P6). The parental lines and progenies were grown open field, in the warm weather conditions of Huelva (Spain), and phenotyped for the fruit skin and flesh color at maturity, while the color information for commercial varieties (CV) was obtained from descriptors of breeder. Skin color descriptors were yellow, green, mottled (yellow or green skin mottled over colored background, Supplementary Figure 1), pale red, red, purple, and black. Flesh color descriptors were white, green, yellow, orange, red, and purple. The fruit color of all materials is presented in Supplementary Data 1, 2.

The DNA of accessions and progenies was isolated from young, fresh, or frozen leaves ground with liquid nitrogen by using the Doyle CTAB method (Doyle and Doyle, 1987). RNA was extracted from the skin of two commercial cultivars with contrasting fruit coloration: “Black Gold” (BG, dark red skin) and “Golden Japan” (GJ, yellow skin). Fruits were collected in triplicates at three different stages: S1 (an immature fruit with green skin), S2 (an intermediate-mature fruit, initial appearance of coloration), and S3 (a maturity stage fruit, full coloration). The fruit skin was separated from the flesh, immediately frozen in liquid nitrogen, and kept at −80°C. Samples were ground in liquid nitrogen, and the RNA was extracted by using the Maxwell RSC Plant RNA Kit (Promega, Madison, WI, USA), and then further DNase-treated by using the TURBO DNA-free Kit (Invitrogen, Carlsbad, CA, USA). Quality and quantity of DNA and RNA were checked in a NanoDrop ND-1000 Spectrophotometer and in 0.8% agarose gels.

### *MYB10* Allele Amplification

A unique primer pair designed in the conserved regions of the *P. persica MYB10.1* (Prupe.3G163100) and *MYB10.2* (Prupe.3G163000) genes was used to genotype all plant materials. Forward (MYB10F2:GTGTGAGAAAAGGAGCTT) and reverse (MYB10NR2:GATATTTGGCTTCAAATAGTTC) primers hybridize in exon 1 (20 bases downstream the start codon of both genes) and in exon 2 (40 bases downstream its start position), respectively (Supplementary Figure 2).

Each PCR reaction contained 1 × NH_4_ and 1.5 mM MgCl_2_ buffers for 1U BioTaq Polymerase (Bioline), 0.2 mM dNTP mix, 0.2 μM of each MYB10F2 (fluorescently labeled) and MYB10NR2 primer, 40 ng of DNA, and MilliQ water to a 10 μl total reaction. PCR conditions were: 94°C for 1 min, 30 cycles of 15 s at 94°C, 15 s at 55°C, and 30 s at 72°C, with a final elongation step of 5 min at 72°C. Amplified fragments were separated by capillary electrophoresis using the ABI Prism 3130xl Genetic Analyzer. The GeneMapper 5.0 software was used to visualize the amplified fragments and precisely size them by correlation to the GeneScan™ 500 LIZ™ size standard.

### SSR Genotyping, Linkage Mapping, and Identification of Haplotypes

Families P1, P2, and P3 were genotyped with LG3 SSR markers (MA039a, UDAp-496, PaCITA10, EPPCU0532, BPPCT007, and BPPCT039). PCR reaction, thermocycler conditions, and capillary electrophoresis were as before, with the annealing temperature adapted to each marker (Supplementary Table 1).

LG3 genetic maps of the pollen and seed parents were constructed by using JoinMap 5.0 (Stam, 1993). For the analysis, each of the multiple bands amplified by MYB10F2/MYB10NR2 was entered as an independent dominant allele. Groups were established with a logarithm of the odds (LOD) score of 3.0, and map distances were calculated with the mapping function of Kosambi (Kosambi, 2016).

To phase the *MYB10* polymorphic alleles into allelic combinations, and therefore into haplotypes (H), we inspected their segregation in the six progenies. Haplotypes for the ABL and CV were inferred with the PHASE v2.1 software (Stephens and Donnelly, 2003; Scheet and Stephens, 2006).

### Amplicon Cloning and Sequencing

All PCR fragments with frequencies higher than 5% in a panel of parental lines were cloned. PCR reactions were purified by using the High Pure PCR Product Purification Kit (Roche, Basel, Switzerland). Amplification was checked in 2% agarose TAE gel, and the amplified fragments were ligated into the Promega pGEM®-T Easy vector and transformed with JM109 *Escherichia coli* cells according to the kit instructions. Colonies were screened by direct colony-PCR using MYB10F2/MYB10NR2 genotyping and capillary electrophoresis. For colonies carrying one fragment of interest, colony-PCR was repeated with common vector T7 and SP6 primers, using the same PCR reaction and thermocycler conditions. PCR products were purified, checked on agarose gel, and sequenced with T7 and SP6 primers by the fluorescent dye termination detection in the ABI 3730 DNA Analyzer.

### Diversity and Phylogeny Analysis

Sequences of the fragments cloned were aligned, vector trimmed, and manually edited by using the Sequencher 5.0 software (Gene Codes Corporation, Ann Arbor, MI, USA). Aligned sequences were imported into Jalview v2 (Waterhouse et al., 2009) for the alignment visualization. Nucleotide basic local alignment search tool (BLASTn) software (Altschul et al., 1990) was run on the individual sequences to find its closest homologous R2R3 MYB TF in the NCBI GenBank database. The same software was used in the GDR webpage (Jung et al., 2019) by using Peach Genome V2.0.1a as the database to find the homologous *P. persica* genomic region. Annotated genes were visualized in JBrowse (Buels et al., 2016). Gene sequences of the closest matches were imported into the Sequencher 5.0 file, with mismatches manually edited. The full contig was exported to MEGA X (Kumar et al., 2018) for the phylogeny analysis. Sequences were clustered by using the UPGMA method (Sneath and Sokal, 1973) using the Tamura–Nei model (Tamura and Nei, 1993) and 500 bootstrap (Felsenstein, 1985) replications. For exon and intron discrimination, cloned alleles were aligned to the R2R3-MYB10 mRNA sequences of *P. persica* (Prupe.3G163100.1)*, P. dulcis* (EU155159.1)*, P. avium* (GU938689.1)*, P. salicina* (KX349091.1)*, Prunus domestica* (EU153580.1), and *P. domestica subsp. insititia* (EU153579.1). DNAsp software v6 (Rozas et al., 2017) was used for the nucleotide diversity analysis, considering gaps. Exonic sequences were translated *in silico* by using ExPASy Translate (Artimo et al., 2012). The resulting amino acid sequences were aligned and compared by using Clustal Omega (Sievers et al., 2011) and visualized in Jalview v2.

### Whole-Genome Sequencing, Gene Cloning, and Analysis of Variability

High-quality DNA isolated from the leaves of two varieties with a contrasting fruit color phenotype was sent to CNAG (Centre Nacional d'Anàlisi Genòmica, Barcelona) for the paired-end library preparation and processed with the Illumina HiSeq 2000 Sequencer. Adapter removal and quality-based trimming of the raw resequencing data were done with Trimmomatic version 0.36 (Bolger et al., 2014). FastQC (http://www.bioinformatics.babraham.ac.uk/projects/fastqc) was used for read quality control before and after trimming. High-quality reads were mapped to the *P. persica* genome version 2.0 (Verde et al., 2017), *P. dulcis* Texas Genome v2.0 (Alioto et al., 2020), and *P. avium* Genome v1.0 (Shirasawa et al., 2017) by using the Burrows–Wheeler Aligner (BWA) (Li and Durbin, 2009), and the resulting alignment files were sorted and filtered by discarding multi-mapped reads. General statistics, depth, and breadth of coverage of sequencing libraries were obtained by using Samtools (Li et al., 2009). Normalized sequencing depth with respect to the mean number of reads of the whole-genome and MYB region was visualized with Circos version 0.69.9 (Krzywinski et al., 2009). The MYB10 region was considered from the first base of 5′ untranslated region (UTR) of the *MYB10.2* gene to the end of *MYB10.3* 3′ UTR. (LG3:18183274–18256025 in peach, chr3: 12147156–12215700 in sweet cherry, and Pd03:15372325–15392346 in almond genomes). The alignment against the *P. persica* and *P. avium* genomes was used to design PCR primers to extend the allelic bands corresponding to the *PsMYB10.1* gene and upstream region. Allele a356 sequence was obtained from the accessions C46 (with H1) and C6 (with H3); a470 from C57 (H2) and C20 (H4), and a243 from C50 (H_i_10/H_i_11). All primers were manually designed based on the sequence read coverage, primer optimal properties, and specificity using the BLASTN function. Primer sequences and annealing temperature used are listed in Supplementary Table 2. Purified PCR products from red and yellow accessions were ligated to a pCR™2.1-TOPO® vector. Ligation reactions were as above with a higher PCR extension time (2 min) for longer fragments. From the whole-gene sequencing, *in silico* translated proteins were aligned and compared for polymorphism identification and to search for the signature motifs conserved in Rosaceae anthocyanin-promoting R2R3-MYB genes. Upstream sequences were aligned and their cis-regulatory motifs were explored by using the PLACE database (Higo et al., 1999).

### Association of MYB10 Alleles and Haplotypes With Fruit Color and Validation in a Panel of CV

The association of the alleles and haplotypes obtained in the 81 ABL with the skin and flesh fruit color was evaluated through a Chi-squared test. Phenotypes were binary classified into presence (for red, purple, and black colors) or absence (for mottled, white, green, yellow, and orange) of anthocyanin-based color on the fruit skin and flesh. The MYB10 primer pair was tested in a panel of 31 commercially cultivated Japanese plum varieties to validate the results (Supplementary Data 2).

### Gene Expression Analysis

The RNA from the fruit skin of two cultivars was reverse transcribed witholigo(dt)20 primer by using the PrimeScript RT-PCR Kit (TaKaRa, Dalian, China). Based on the results of the previous full-gene sequencing, primers M101_RT_F and M101_RT_R were designed to amplify and sequence the whole coding sequence of the alleles a356 and a470 (Supplementary Table 2). The specificity of the primers was tested by PCR using genomic DNA from the two cultivars. Later, PCR with complementary DNA (cDNA) as a template was used to check the expression of a356 and a470. Expression of the MON reference gene was used as a positive control (Kim et al., 2015). Amplification products were purified and sequenced by using the same primer pair. The results were imported into Sequencher 5.0 and aligned to full-gene sequences of a356 and a470 for their identification. Sequencing results were incorporated into the previously created contig containing messenger RNA (mRNA) sequences.

## Results

### *MYB10* Homology Analysis and Allele Mapping

To amplify and isolate *PsMYB10* allelic bands, we used the PCR primers designed in the conserved regions of *PpMYB10.1, PpMYB10.2*, and *PpMYB10.3*. In a panel of 81 Japanese plum ABL, we identified 16 alleles with size ranging from 243 bp to 500 bp, with only (a466) being monomorphic. In all, the minimum number of alleles observed per genotype was three and the maximum was eight, with a mean of 5.7 alleles, indicating the amplification of more than three *MYB10* loci for a diploid species.

**Figure 1:**
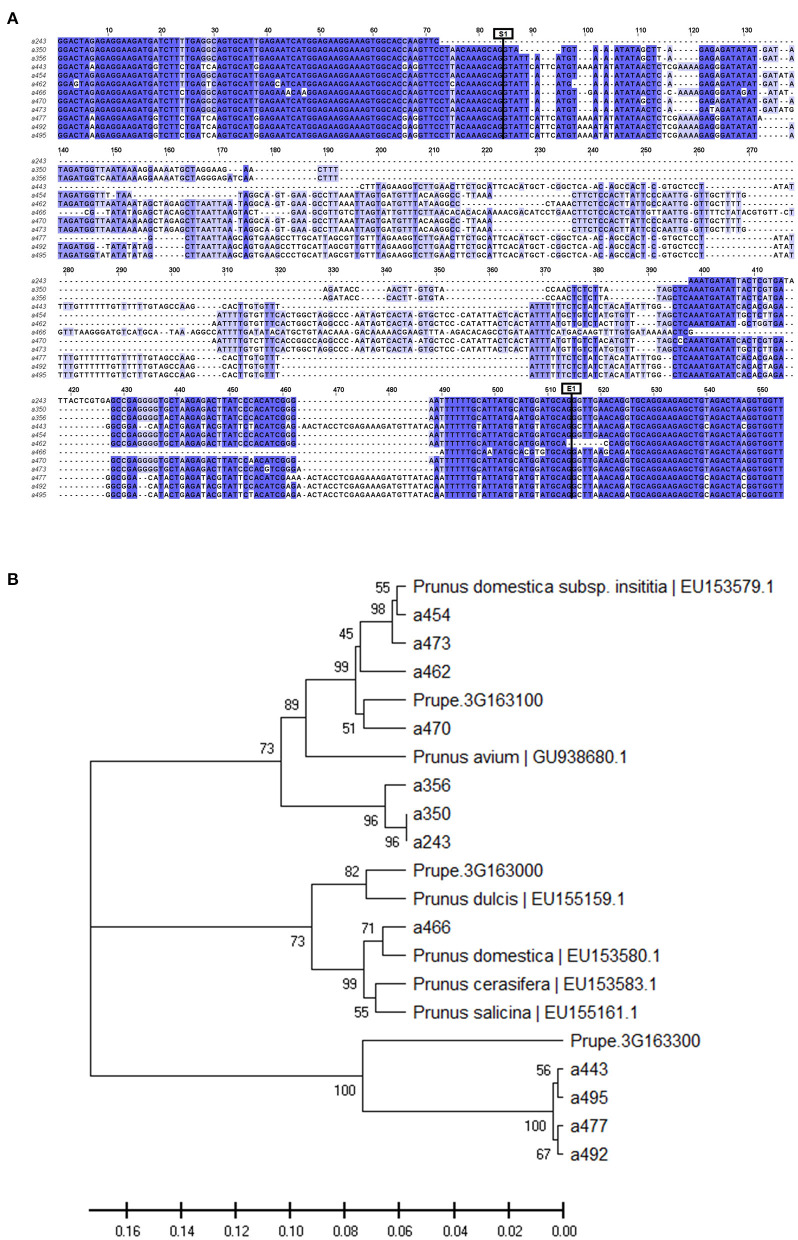
**(A)** View of the alignment of the 12 cloned allele sequences (a243 to a495). Intron starts at position 85 (S1) and ends at position 514 (E1). **(B)** Phylogeny tree grouping the cloned sequences with other MYB10 *Prunus* sequences from the NCBI database and the genes of peach *MYB10.1* (Prupe.3G163100), *MYB10.2* (Prupe.3G163000), and *MYB10.3* (Prupe.3G163300).

The alleles with a frequency above 5% (12 in total including the monomorphic a466) were cloned and sequenced. Allele sequence alignment identified SNPs as well as indels. Nucleotide diversity was π = 0.204, with most variations in the intronic region (π_Exon_ = 0.071 vs. π_Intron_ = 0.244) (Figure 1A, Supplementary Data 3).

The BLAST analysis of these sequences against *P. persica* and the other *Prunus* sequences available in the NCBI database showed the best hit of all of them with *MYB10* genes (Supplementary Table 3). Seven (a243, a350, a356, a454, a462, a470, and a473) were homologous to the peach *PpMYB10.1* gene sequence (Prupe.3G163100), with the closest homology to the *P. domestica subsp. insititia* MYB10 (*PiMYB10*) gene sequence EU153579.1 (Supplemental Table 3). Only the monomorphic a466 allele was homologous to *PpMYB10.2* (Prupe.3G163000), with high homology to the *P. domestica* MYB10 (*PdmMYB10*) gene sequence EU153580.1. The four remaining alleles (a443, a477, a492, and a495) were homologous to *PpMYB10.3* (Prupe.3G163300), with the closest homology to the *P. avium MYB10* (*PaMYBA1*) gene sequence EU153580.1.

Consistent with the BLAST results, the phylogeny analysis of the amplified alleles together with orthologous *Prunus MYB10* genes identified three groups in agreement with the peach *PpMYB10.1, PpMYB10.2*, and *PpMYB10.3* gene organization (Figure 1B). The seven alleles homologous to *PpMYB10*.1 were clustered with the *PiMYB10* gene sequence EU153579.1 and with the *PaMYB10* gene sequence GU938680.1. The monomorphic allele homologous to *PpMYB10.2* was clustered with the *MYB10* sequences of *P. dulcis* (EU155159.1), *P. domestica* (EU153580.1), *Prunus cerasifera* (EU153583.1), and *P. salicina* (EU155161.1). However, the four alleles homologous to *PpMYB10.3* were clustered only with this peach gene, in contrast to what was expected from the BLAST analysis where best hits were found with *P. avium*. This discrepancy is attributable to the low query coverage of these alleles in the BLAST analysis (≤62%) (Supplementary Table 3).

**Figure 2:**
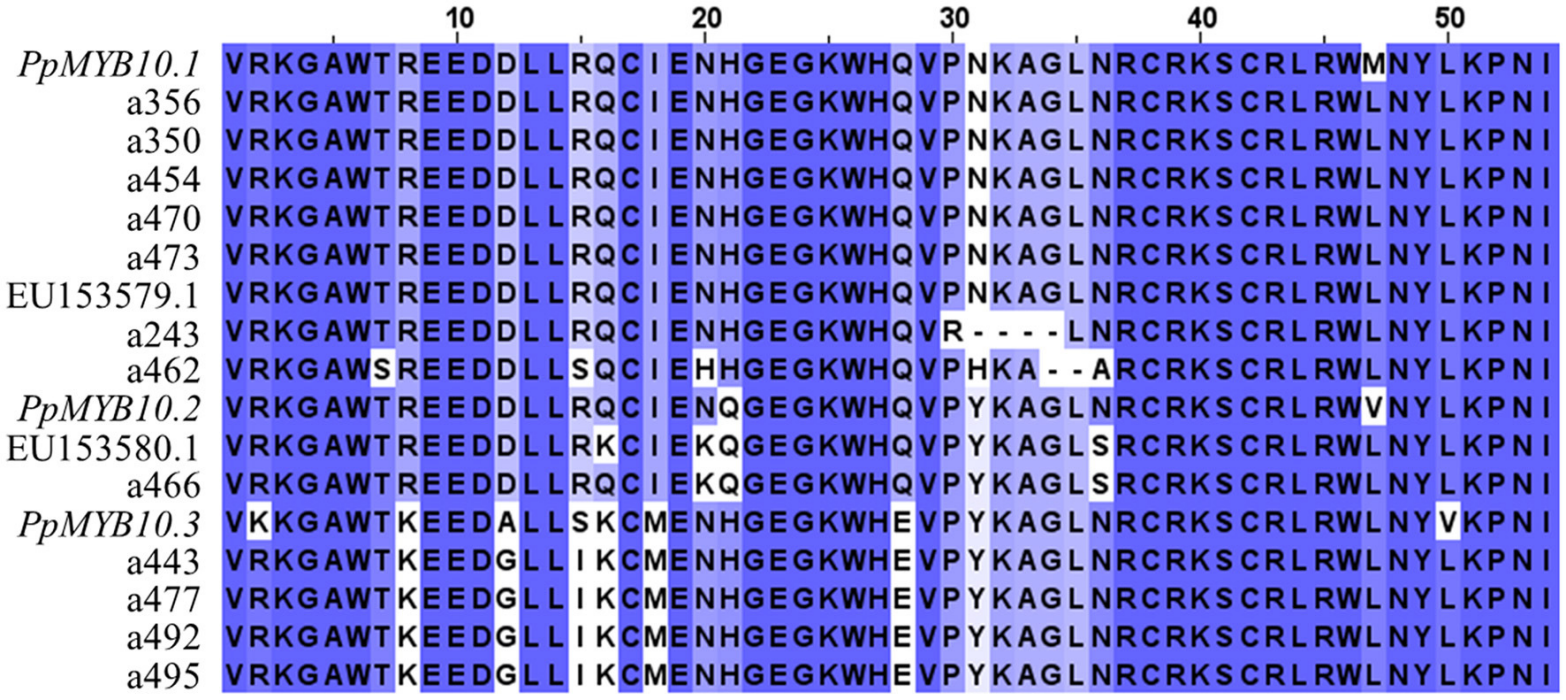
Deduced amino acid sequence of the cloned alleles (a243 to a495) compared to the peach PpMYB10, *Prunus domestica subsp. insititia* (EU153579.1) and *P. domestica* (EU153580.1) R2R3-MYB10 translated sequence.

Nucleotide diversity was higher across the *MYB10.1* allele than the *MYB10.3* allele (π = 0.066 and π = 0.007, respectively) (Figure 1A, Supplementary Data 3). The deduced amino acid sequence revealed that all alleles encoded a peptide, which included a characteristic R2 domain (Figure 2). Changes were observed in 19 out of 54 amino acid positions, including deletions and conservative substitutions. A large number of variants in the exons were synonymous, with average *Ka/Ks* = 0.319 for the 66 allele pair comparisons. Amino acid sequences of *PiMYB10*, a350, a356, a454, a470, and a473, were identical and shared 98.15% identity to *PpMYB10.1*, while the other *MYB10.1* alleles, a243 and a462, shared 98 and 90.38% sequence identity, respectively, with the previous group (Supplementary Data 3). The amino acid sequence of a466, homologous to *PpMYB10.2*, was closer to *P. domestica* (98.15% identity) than to the peach (94.44%) while the remaining alleles shared 100% protein identity between them and 92.59% compared to the *PpMYB10.3*.

To confirm that the alleles corresponding to the LG3 MYB10 genes, three families (P1, P2, and P3) were genotyped with the MYB10 primer pair plus five LG3 SSR markers. All alleles mapped between the markers MA039a and/or UDAp-496 and PaCITA10 (Supplementary Figure 3), which are in agreement with the position of the peach *MYB10* gene cluster.

### Assignation of MYB Alleles Into Haplotypes

**Figure 3:**
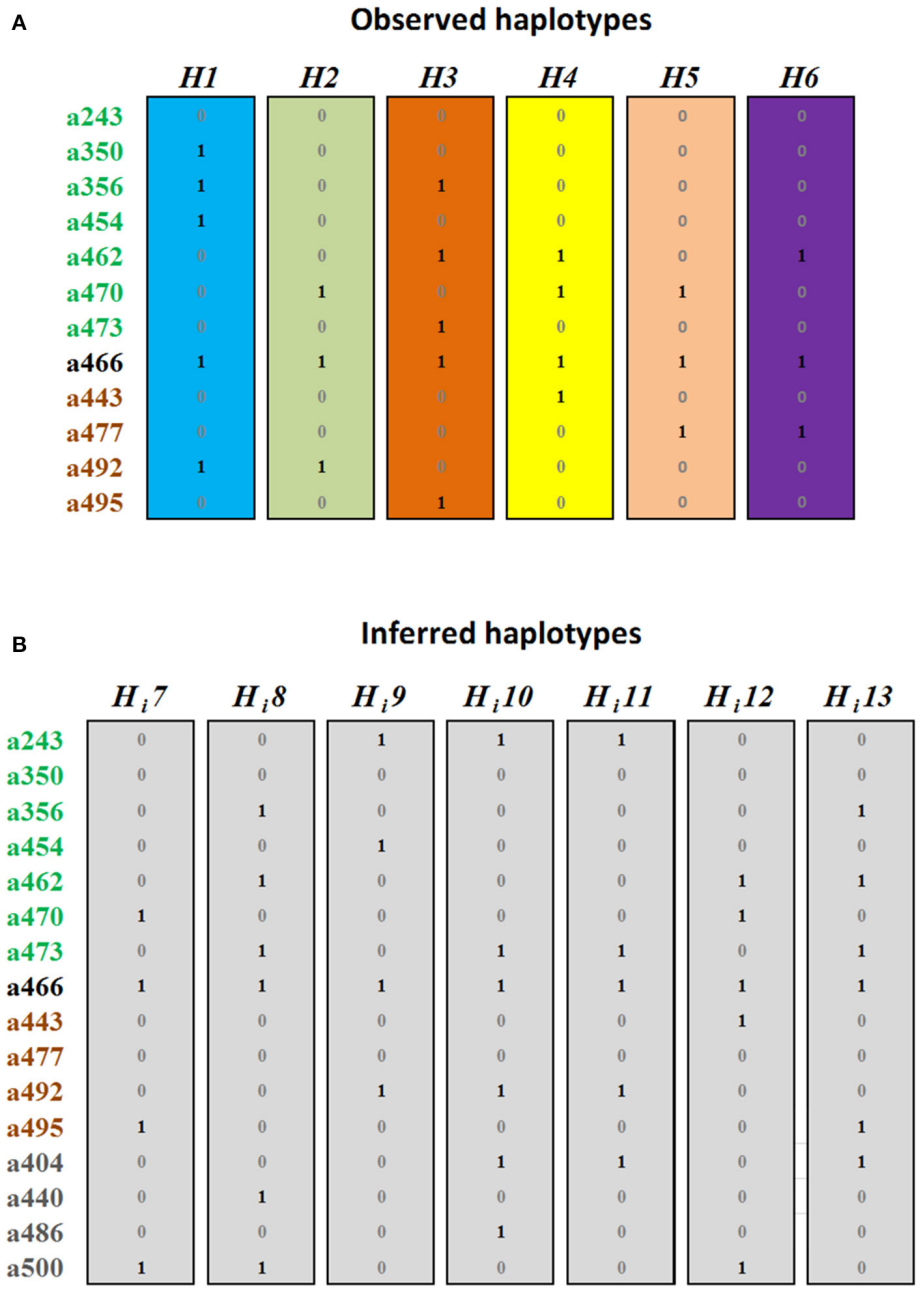
Haplotypes with (1) or without (0) each *MYB10* allele, grouped and colored according to their homology with *PpMYB10.1, PpMYB10.2*, and *PpMYB10.3*. **(A)** Haplotypes identified in six F1 progenies. **(B)** Haplotypes inferred from the collections of advanced breeding lines (ABL) (H_i_7–H_i_12) and commercial varieties (CV) (H_i_12, H_i_13).

Considering that two alleles segregating in phase cannot be allelic of the same gene, we evaluated the segregation of the alleles in 382 individuals of 6 biparental families. We identified six allele combinations segregating in phase, i.e., determining haplotypes (H). Sorting the alleles by their similarity with the *PpMYB10.1-3* genes and size, these were H1 with the alleles a350-a356-a454-a466-a492, shared by C4, C5, C8, C9, and C26; H2 with the alleles a470-a466-a492, shared by C4, C8, C19, C26, and C66; H3 with a356-a462-a473-a466-a495, shared by C6 in homozygosis and by C5, C11, C14, and C66; H4 with alleles a462-a470-a466-a443, shared by C14 and C19; H5 with a470-a466-a477, in C9; and H6 with a462-a466-a477, in C11 (Figure 3A, Supplementary Figure 4).

These six haplotypes explained the genotype of 74 (91.36%) of the ABL, while five additional inferred haplotypes (H_i_7–11) were required to explain the genotype of the seven remaining ABL (Figure 3B). In six of them (7.41%), the H_i_ was found to be in combination with an observed H, while C50 was unique with the two haplotypes inferred. All H_i_ contained one or several of the alleles with a frequency ≤5%, and were, therefore, discarded for cloning, sequencing, and homology analysis (haplotype combinations for all ABL are shown in Supplementary Data 1).

The combination of haplotype and allele homology data revealed that the haplotypes H1, H3, and H_i_8 contain three *MYB10.1* alleles; haplotypes H4, H_i_9, H_i_10, and H_i_11 contain two alleles, and H2, H5, H6, and Hi7 contain only one allele. This indicates multiple copies of *MYB10.1*, at least in some Japanese plum cultivars. By contrast, only one *MYB10.2* and one *MYB10.3* allele per haplotype were identified.

### High-Throughput Sequence Analysis of the *MYB10* Region

**Figure 4:**
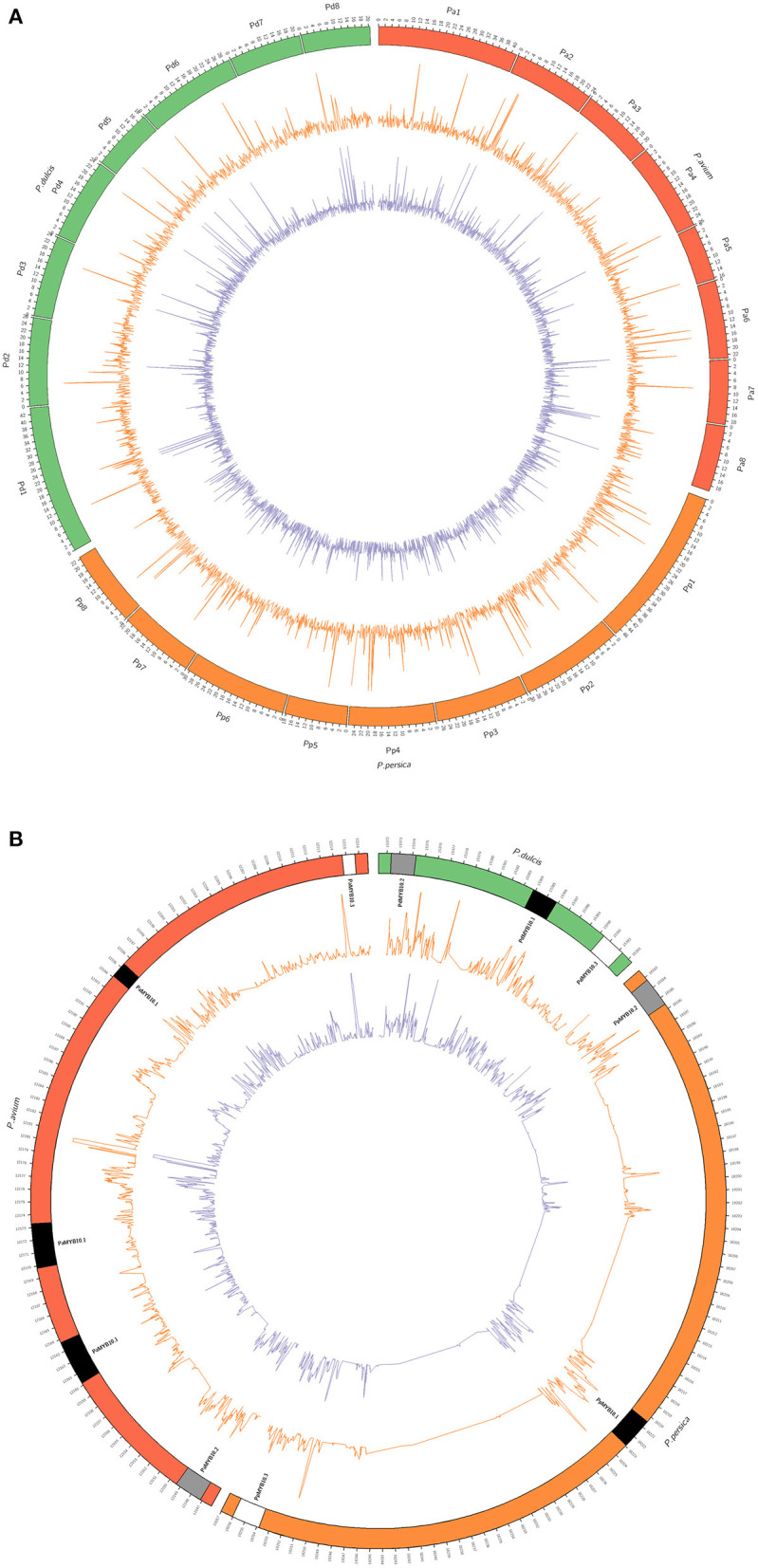
Circular plots of the sequencing depth of C20 (orange line) and C46 (purple line) alignments against the genomic reference sequences of *Prunus avium* (Shirasawa et al., 2017), *Prunus persica* (Verde et al., 2017), and *Prunus dulcis* (Alioto et al., 2020). **(A)** Whole-genome alignment (genomic units in megabases) and **(B)** alignment of the chromosome 3 *MYB10* region (genomic units in kilobases). *MYB10* genes (*MYB10.1* in black, *MYB10.2* in gray, and *MYB10.3* in white) are represented in their corresponding genomic position for each reference species. Depth was normalized for a better visualization and sequence comparison.

The accessions C20, with yellow skin and flesh and C46, with red skin and flesh, were selected for whole-genome sequencing. The number of reads obtained was 34.671 M for C20 and 117.115 M for C46. Reads were mapped against the reference genomes of peach (Verde et al., 2017), almond (Alioto et al., 2020), and sweet cherry (Shirasawa et al., 2017). For each of the two cultivars, the whole genome as well as along LG3, the depth was similar for all three alignments (Supplementary Table 4, Figure 4); however, it varied considerably along the *MYB10* region depending on the species genome. Such differences were observed in both genic and intergenic regions. Depth in the *MYB10* region was higher than that of the observed genome width when aligned against a peach and an almond, mainly due to a high number of reads aligned in the genic regions (1.7 times higher in C20 and 1.8 times in C46). In contrast, depth in the *MYB10* region was lower, both in the genic and the intergenic regions, when aligned against a sweet cherry. The breadth of coverage was also higher in the MYB10 region than genome width in the peach and almond alignments, while the opposite was observed when aligned against a sweet cherry. This data (higher depth in a high breadth coverage scenario) are consistent with a higher number of copies of the *MYB10* genes in C20 and C46 compared with the peach and almond reference genomes.

### Gene cloning

Whole-genome sequence alignments against the peach and almond reference sequences were used to design primers to fully amplify *PsMYB10.1, PsMYB10.2*, and *PsMYB10.3* alleles in the accessions selected according to their haplotypes. As a result, we obtained the whole-gene sequence corresponding to the a356 allele in two haplotypes (a356_H1_ and a356_H3_), to the a470 allele also in two haplotypes (a470_H2_ and a470_H4_), as well as the a466_H1_ and a492_H1_ alleles. The remaining alleles could not be isolated with the primers designed for *PsMYB10.1*.

**Figure 5:**
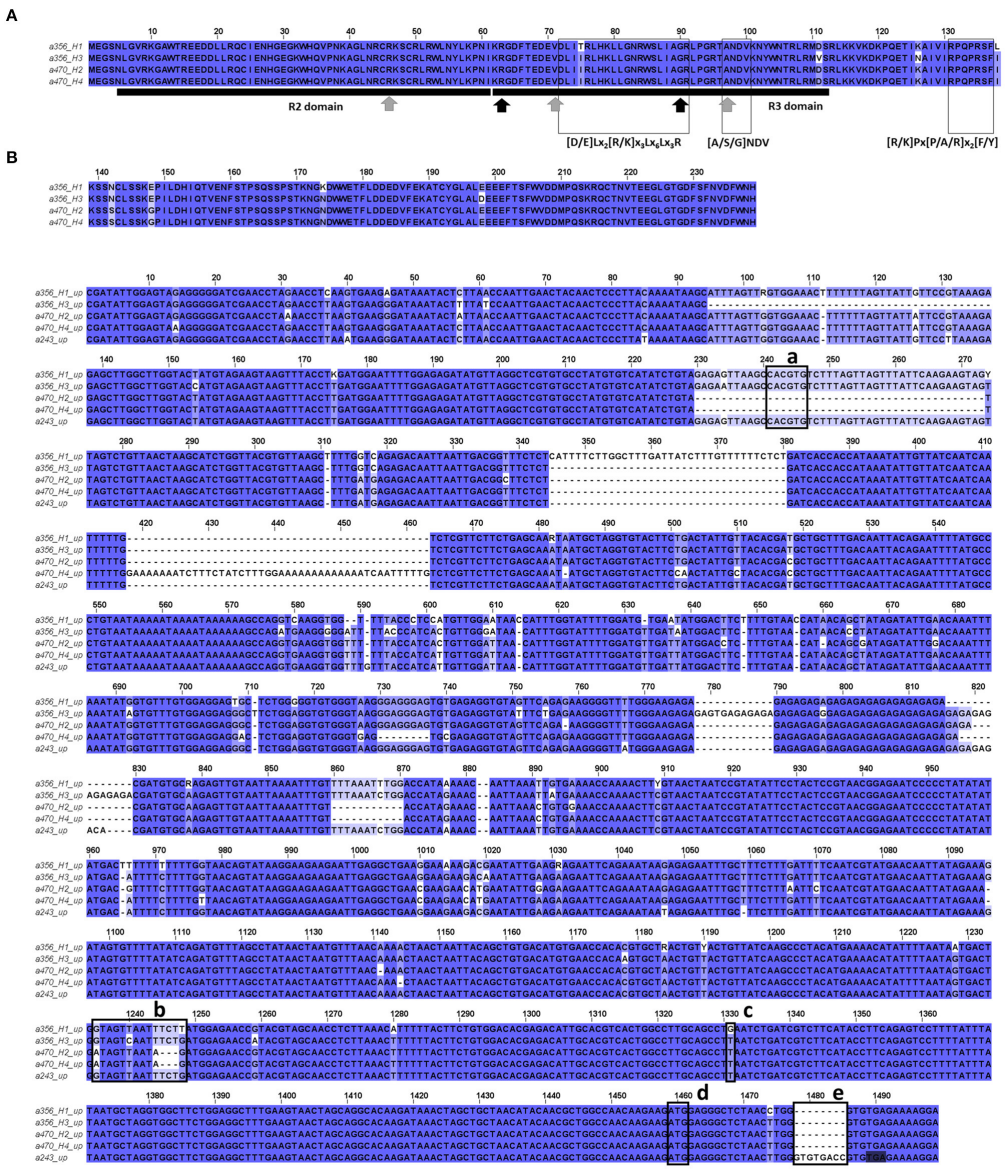
**(A)** Alignment of *PsMYB10.1 in silico* translated proteins from a whole-gene sequence. Conserved amino acids (gray arrows) and signature motifs ([A/S/G]NDV; [R/K]Px[P/A/R]x2[F/Y]) from R2R3-MYB anthocyanin-promoting proteins from Rosaceae were detected in all PsMYB10.1 sequences (Lin-Wang et al., 2010). Conserved motif [D/E]Lx2[R/K]x3Lx6Lx3R indicates a possible interaction with basic-helix-loop-helix (bHLH) proteins. Black arrows indicate key residues in *PpMYB10.1* function (Zhou et al., 2018). **(B)** Polymorphisms found between a356_H1_, a356_H3_, a470_H2_, a470_H4_, and a243 upstream sequences: (a) G-box motif in the 44 bp indel; (b) polymorphisms in the R1-motif (Espley et al., 2009); (c) SNP S3_12879559 (Salazar et al., 2017); and (e) 8 bp insertion in exon 1 of a243, 4 bases before an in-frame STOP codon (shadowed). The start codon is marked as (d).

Amplicon size of a356_H1_, a470_H2_, and 470_H4_ was 1.5 kb while that of a356_H3_ was ca. 3 kb due to a larger intron 2 (Supplementary Data 4). Their CDS encoded a 239 amino acid protein. Amino acid changes were observed at eight positions, with two at R3 but none at R2 (Figure 5A). While both a470 alleles were identical, six amino acid substitutions were found between the two a356 alleles, four with residues with biologic similarity. Motifs and amino acids conserved among Rosaceae anthocyanin-promoting R2R3-MYB genes, described in Lin-Wang et al. (2010) and Zhou et al. (2018), were detected in all a356 and a470 sequences. Amplicon size of a466 and a492 was 2 and 3 kb and encoded the proteins of 243 and 160 amino acids, respectively.

We took advantage of a high homology between the Japanese plum and a sweet cherry at the intergenic MYB10 regions to amplify and sequence the upstream regulatory region of the *PsMYB10.1* gene. We obtained up to 1,850 bp upstream alleles a243_Hi10_, a356_H1_, a356_H3_, a470_H2_, and a470_H4_. The sequences revealed additional polymorphisms (Figure 5B), among them, an insertion of 8 bp in exon 1 of a243 before the hybridization site of the primer initially used to obtain the short *PsMYB10* allelic bands (MYB10F2) leading to a STOP codon 4 bp after the insertion. In addition, we identified, in a356 and a243 contigs, a 44 bp insertion containing a G-box binding motif 1,208 bp upstream of the gene start codon, and two polymorphisms in the R1-motif (described in Espley et al., 2009) of a470 with an SNP and a 5 bp polymorphism.

We identified the polymorphism described by Salazar et al. (2017) (SNP S3_12879559) 128 bp upstream of the start codon of *MYB10.1*. In this position, all the alleles except a356_H1_ carried the nucleotide T.

### Haplotypes Associated With Skin Color

In the ABL collection, 65.4 and 38.2% of the accessions produced fruits with anthocyanin colors in the skin or flesh, respectively (Supplementary Figure 5). The 11 polymorphic alleles with a frequency higher than 5% were tested for association with skin and flesh color traits in this collection through a Chi-squared test. The allele showing the highest association with skin color was a356 (*p* = 1.96 × 10^−18^) (Supplementary Table 5). This allele was absent in all varieties with yellow or green skin but present in 52 out of 53 skin-colored ones. No alleles were found to be associated with fruit flesh color.

Allele a356 was present in haplotypes H1, H3, and H_i_8 in the ABL collection (Figure 3); all accessions bearing skin-colored fruits but one (C74; H2/H6) had one or two copies of these haplotypes (Supplementary Data 1, Supplementary Figure 6), while those lacking anthocyanin pigments in the skin combined two copies of any of H2, H4, H5, H_i_10, or H_i_11. Consistently, most progenies containing either H1 or H3 had colored fruits (95.90 and 94.90% of cases, respectively), while those H2/H4 or H2/H2 fell mainly (95.12 and 95.24%, respectively) within the anthocyanin-less category, in particular with the mottled phenotype. In agreement with this, all ABL with the mottled phenotype had H2. In the progenies, combinations between H2 and H6 were either mottled (26.67%) or red (73.33%).

Apart from missing a356, only haplotypes H2, H4, and H5 amplified a470. The phase information, as well as phylogenetic analysis, are compatible with a356 and a470 being allelic of the same *MYB10.1* gene (from now on *PsMYB10.1a*). Therefore, *PsMYB10.1a* may have, at least, a356 and a470 as alleles, with a356 associated with anthocyanin color and a470 with its absence.

To validate the association, we analyzed a collection of 31 CV. Two new haplotypes were needed to explain the genotypes of “Sunkiss” (H4/H_i_12) and “Gaia” (H5/H_i_13), with H_i_13 bearing a356 and conferring coloration (Figure 3). The marker was able to correctly predict the presence or absence of red color in the fruit skin in all cases: all colored cultivars carried at least one haplotype with the a356 allele while the rest had green or yellow fruits. In ABL and progenies, the H2 haplotype, in combination with other anthocyanin-less associated haplotypes, was observed in mottled fruits. This could not be validated in a more diverse collection since none of the CV showed this phenotype (Supplementary Data 2).

To investigate the C74 (H2/H6) outlier, we used the primer pair M101_f/r to identify possible null alleles in this ABL. The 1.5 kb sequence revealed a new *MYB10.1* allele identical to a470 except for a four-nucleotide deletion in the first intron (Supplementary Data 4). This allele (a467) was hidden by the monomorphic a466 in the electrophoresis profile. Using specific primers in all the germplasm, we found the a467 allele in 16 ABL and 6 CV, all with H6 or H2. While all genotypes with H6 had a467, only 13 of the accessions with H2 (27.08%) had this allele. Since C72 and C74 are H2/H6, it is uncertain whether a467 was in heterozygosis (carried in H6 only) or in homozygosis (carried in both H2 and H6). The segregation of a467 in P6 (with C11; H3/H6) confirmed that this allele co-segregates within H6, and therefore corresponds to an LG3 *MYB10* gene (Supplementary Figure 3).

### Expression of *PsMYB10.1a* in the Skin

**Figure 6:**
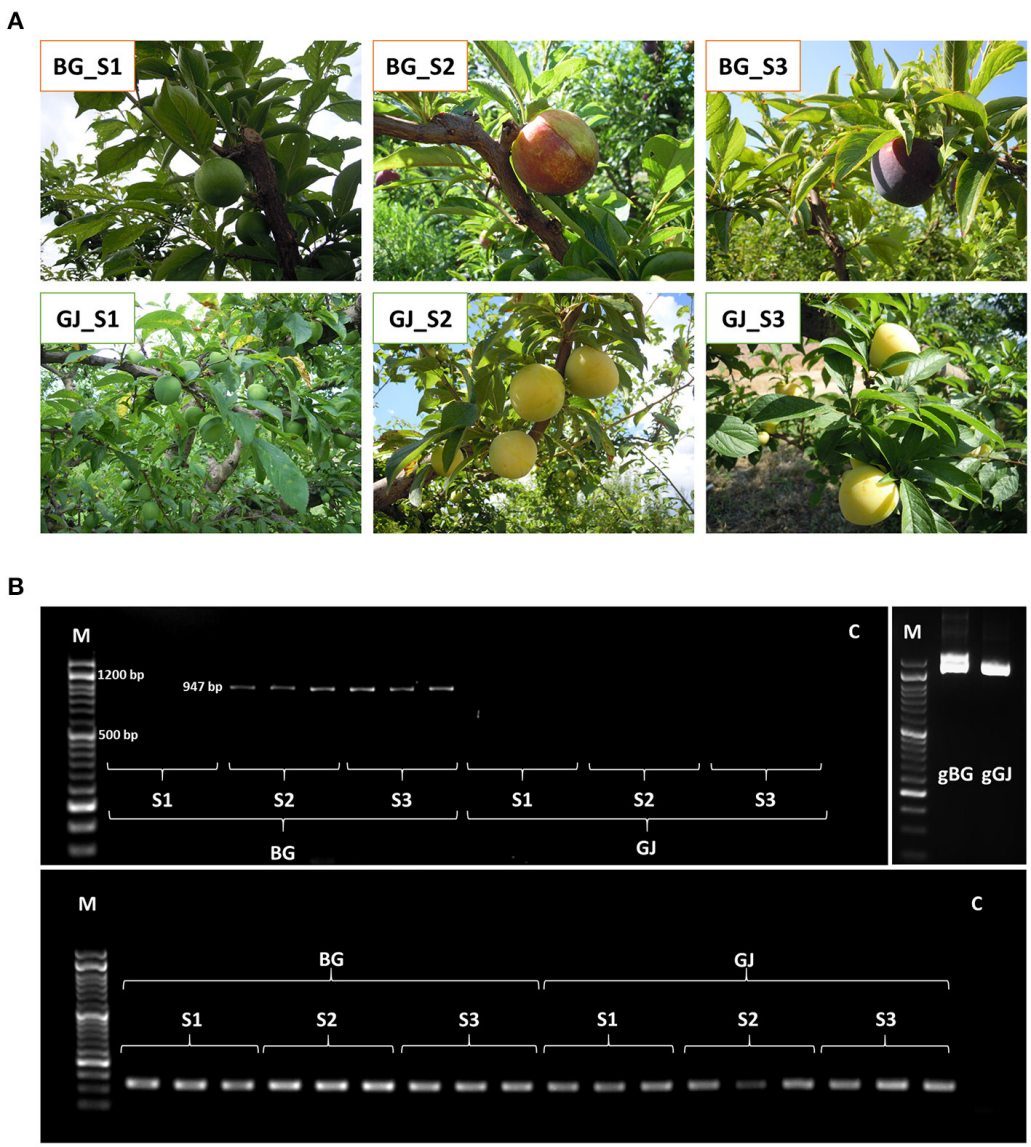
**(A)** RNA was extracted from the skin of Black Gold (BG) and Golden Japan (GJ) at three maturation stages (S1, S2, and S3). **(B)** Top row left: *PsMYB10.1* amplification from complementary DNA (cDNA) samples (BG_S2 and BG_S3). Band size 947 bp; top row right: *PsMYB10.1* amplification of genomic DNA (BG and gGJ). Band size 1.5 kb; bottom row: expression of the *MON* reference gene in all cDNA samples tested. Well M: DNA Ladder 50 bp ready-to-use (GeneON); well C: no template control.

The expression of the *PsMYB10.1a* alleles a356 and a470 in the fruit skin was evaluated in the black “BG” (H1/H2) and yellow “GJ” (H4/H_i_9) varieties, at different stages of fruit development. A band with the expected size was obtained in the S2 and S3 stages of BG (Figure 6). Band sequencing revealed that this band corresponded exclusively to the a356 allele. No band was observed in GJ cDNA, indicating that a470 was not expressed in BG or in GJ. The alignment of a356-mRNA to different R2R3-MYB10s from databases detected a shared intron splicing mechanism (data not shown).

## Discussion

Major genes and QTLs for the traits related to anthocyanin coloration have been mapped along with the eight linkage groups of *Prunus* species (see summary in Supplementary Table 6). Several studies have demonstrated a major role of R2R3-MYB genes in the transcriptional control of the accumulation of anthocyanins in rosaceous crops. In particular, the expression of R2R3-MYB10 genes in fruit tissues has been correlated with the transcription of anthocyanin structural genes and pigment accumulation. The peach genome contains six R2R3-MYB10 genes, three of them in LG3, and three in LG6. Two of the LG6 genes (about 60 kb apart) co-localize with a Leaf color trait (Howad et al., 2005), while those in an LG3 (*PpMYB10.1, PpMYB10.2*, and *PpMYB10.3*) cluster in a genomic region of ca.70 kb co-localizing with an anther color gene (*Ag*) (Arús et al., 1994; Rahim et al., 2014). *PpMYB10.1* and *PpMYB10.3*, annotated as “Anthocyanin regulatory C1 protein” [AnnoMine gene description; PLAZA4.0 (Van Bel et al., 2018)], are expressed in fruit tissues at levels correlating with the accumulation of anthocyanins in the skin (Rahim et al., 2014). In Japanese plum, the sustained increase in the expression of *PsMYB10* during fruit ripening was in correlation with the accumulation of anthocyanins, suggesting the involvement of *PsMYB10* in the regulation of the transcriptional control during the biosynthesis of anthocyanins (González et al., 2016b). Considering that the modulation of MYB genes (including changes in either the promoter or their genic region) is enough to produce changes in the accumulation of anthocyanins (Ban et al., 2007) and that Salazar et al. (2017) identified the LG3 QTL for the skin color in Japanese plum, it is reasonable to consider that the genetic variation in *PsMYB10* is involved in the observed variation in fruit color. This motivated our search for polymorphisms in the Japanese plum LG3 R2R3-MYB genes to explain the observed variability in fruit color.

While, only a few *R2R3-MYB10* genes have been sequenced in Japanese plum, this gene family has been well characterized in other plant species, where systematic functional characterization identified highly conserved amino acid motifs and a characteristic *R2R3-MYB* DNA-binding domain (Kranz et al., 1998; Stracke et al., 2001; Zhang et al., 2020) with the homology values higher than 40% (Stracke et al., 2001). In this study, we took advantage of the high domain conservation and the high homology between a peach and Japanese plum (Mnejja et al., 2004) to obtain the partial sequence of 16 Japanese plum *MYB10* alleles. These alleles were amplified in a panel of Japanese plum accessions in numbers higher than the maximum expected for three loci in a diploid genome, confirmed through the progeny and haplotype segregation analysis, suggesting the gene duplication. The segregation of the bands in six biparental families allowed the definition of six haplotypes, each with three to five alleles. Combinations of these haplotypes were able to explain the genotype of most of the ABL and CV studied here, while the inference of additional haplotypes was needed to fully explain the genetic variability. Overall, this revealed high variability in this region, and high allelic variability (π = 0.204) when considering all alleles with a frequency >5% (12 in total), especially in the intronic regions.

Lin-Wang et al. (2010) isolated the R2R3-MYB10 homologs from cDNA of the major rosaceous crops. Here, we found some closely matched 12 alleles amplified at a frequency higher than 5%, grouping in three clusters in agreement with three tandemly duplicated *PpMYB10* genes on LG3. Most of the alleles (7) clustered with the *PpMYB10.1* gene, contrasting with the phylogeny observed by Lin-Wang et al. (2010), where all but two of the isolated MYB *Prunus* alleles clustered with *PpMYB10.2*, as further confirmed for *PsMYB10* and *ParMYB10* genes by Rahim et al. (2014). Unlike Lin-Wang et al. (2010), by isolating the alleles from genomic DNA, we could identify a large number of *MYB10.1* alleles independent of their expression. On account of the high homology between the *MYB10* genes, our strategy allowed us to assign some of the bands to gene alleles. In addition, the new grouping of previously isolated alleles will help to identify new candidate genes for fruit color as well as the further assignment of sequences to alleles or to novel genes in other *Prunus* species.

Haplotype reconstruction, homology, and phylogeny data suggested more than one *MYB10.1* gene in at least some *P. salicina* genotypes. Gene duplication is highly concordant with the number of alleles amplified in H1, H3, and H_i_8 (with three alleles homologous to MYB10.1) and in H4, H_i_9, H_i_10, and H_i_11 (with two alleles homologous to MYB10.1). Unlike *PsMYB10.1*, all haplotypes had a single copy of *PsMYB10.2* (with the monomorphic allele a466) and *PsMYB10.3* (either a443, a477, a492, or a495). Similarly, sweet cherry (Shirasawa et al., 2017) and apricot (Jiang et al., 2019a) genomes have more than three *MYB10* genes in the region. Although a fully assembled genome of *P. domestica* is not yet available, we detected a *MYB10.1* triplication in the scaffolds of its draft genome v1.0.a1 (data not shown) (https://www.rosaceae.org/species/prunus_domestica/genome_v1.0.a1). It is noticeable that not all haplotypes (observed or inferred here) carry the same number of *PsMYB10.1* alleles. This may be due to (i) a different number of LG3-*MYB10* copies in Japanese plum cultivars, (ii) a null allele due to mis-amplification or to same migration of different alleles of the same size in electrophoresis analysis, or (iii) a combination of (i) and (ii). The first hypothesis is highly possible considering the complex hybridization history of Japanese plum genotypes. Cultivated Japanese plums are complex interspecific hybrids between *P. salicina* and other species such as *Prunus simonii, P. cerasifera*, and American plums, with each cultivar having a different degree of these genomes in its genetic background, depending on the breeding history (Okie and Ramming, 1999; Boonprakob et al., 2001). In fact, individuals from a single species may have different genome sizes and genetic compositions, which fall within the concept of the pan-genome, i.e., all individuals within a species may have a core set of shared genes (pan-genome) plus a set of genes, similar to the insertion/deletion of genic copy number variants (CNVs) shared by only some individuals. Gene duplication, and in general gene CNV and the presence/absence of variants (PAV), is a frequently observed phenomenon in plants and is usually associated with both domestication and post-domestication diversification (Lye and Purugganan, 2019). In *Prunus*, the genes related to some quantitative traits are in hotspots (da Silva Linge et al., 2021), in certain cases located in clusters of duplicated genes (Bielenberg et al., 2004; Wells et al., 2015; Gu et al., 2016; da Silva Linge et al., 2021). Gene duplications can increase the gene product and result in altered patterns of gene expression (Innan and Kondrashov, 2010), which could explain the variability in the plum fruit color, including the mottled phenotype. A *de novo* sequence of *P. salicina* together with the sequence of a panel of cultivars will help to elucidate the impact of gene duplications in its phenotypic variation, especially in fruit color. Very recently, the *de novo* sequence of two *P. salicina* cultivars, “Zhongli No.6” (https://www.rosaceae.org/Analysis/9019655) and “Sanyueli” (Fang et al., 2020), has been released. These two sequences are still in their first version, with 318 Mb for “Zhongli No.6” and 284 Mb for “Sanyueli,” which are ca. 53 and 19 Mb larger, respectively, than expected considering the size and homology with the peach genome (265 Mb). The alignments with both genomes of C20 and C46 Illumina sequences together with the BLAST analysis of the MYB alleles obtained here (data not shown) highlight the complexity of the region: the C20 and C46 sequences aligned in two MYB10 regions, 2 Mb apart, in the LG3 of the “Zhongli No.6” genome, while gaps and misalignments were revealed when mapped against the “Sanyueli” genome. The authors refer to these cultivars as Chinese plum and their interspecific hybridization history is not reported, so their kinship with the panel of varieties studied here (used in occidental breeding programs) is unknown.

The simplicity of the electrophoretic analysis that we used hides allelic complexity that can be revealed with the allele cloning and sequencing. We have proven this for the two alleles a356 and a466. In the first case, we observed polymorphisms in the upstream regulatory region of a356 in H1 and H3, although both alleles are equally associated with anthocyanin color. For a466, we isolated two alleles of almost the same size migrating together on electrophoresis, corresponding to *PsMYB10.1* (a467) and *PsMYB10.2* (a466). Likewise, as two duplicated genes may share alleles of equal size, resulting in a PCR band akin to a unique allele, we cannot discard the duplication of *PsMYB10.2* or *PsMYB10.3*. This emphasizes the utility of using haplotypes for the MAS since recombination in the region is unlikely.

The number of haplotypes is high when taking into account the expected reduced size of the region (73 kb in peach, 20 kb in almond, and 69 kb in sweet cherry genomes), which can be explained by the variability of the alleles and the complex hybridization history of Japanese plum. In *Prunus*, the size of this region varies independently of the number of *MYB10* genes, the intergenic regions being shorter in a sweet cherry and an almond (14.2 kb on average in a sweet cherry and 7.0 kb in an almond, compared to 32.8 kb in a peach). Such a high homology between the MYB genes, together with the unlikely recombination in the region, makes it difficult to assign the MYB alleles to their corresponding genes. Long-read DNA sequencing of the LG3-MYB10 region in cultivars with different haplotypes should provide valuable data such as gene number and allelic assignment, together with gene order, the distance between them, and the size of the region.

Whole-genome sequence alignment of C20 and C46 against the peach reference genome found a low-sequence coverage in the intergenic *MYB10* sequences (<0.30), while it was considerably higher in the genic regions (>0.95). The alignment with the sweet cherry genome provided better coverage in the intergenic regions (>0.47). This highlights the importance of a high-quality reference sequence for highly complex regions, containing duplicated genes.

Primers were designed by sequence alignment to fully amplify the a356 and a470 alleles from different haplotypes. Their predicted amino acid sequence contained motifs conserved in other anthocyanin-promoting R2R3-MYBs of the Rosaceae family as well as an intact bHLH domain (Lin-Wang et al., 2010). No polymorphisms were found in the protein sequences that could explain a possible loss of function, including the two key amino acids necessary for the *PpMYB10.1* function identified by Zhou et al. (2018), which were unaltered in our sequences.

Our data suggest that a356 and a470 are allelic of *PsMYB10.1a*, with a356 being associated with the presence of anthocyanins in the fruit skin, and expressed in advanced stages of red fruits only, unlike a470. González et al. (2016a) sequenced the total RNA from the skin of “Angeleno” (H1/H3; dark skin) and “Lamoon” (unknown MYB10 genotype; yellow skin). Allele a356 was expressed in “Angeleno” only, while no a470 reads were recovered (data not shown) confirming a possible loss of function of *MYB10.1a*-a470. This could be explained by the deletion of a G-box sequence in its upstream region. In Arabidopsis, the light-induced *HY5* enhances the expression of the *MYB* ortholog *PAP1* (the production of anthocyanin pigment 1) by binding to a G-box motif (CACGTG) in its promoter (Shin et al., 2013). This mechanism is conserved and has been described in an apple (An et al., 2017). Therefore, the deletion of this motif may prevent the binding of expression enhancers. Another cause of a470 loss of function could be the polymorphisms observed in the R1 motif, which has functional implications in *MYB10* expression. Espley et al. (2009) described multiple tandem copies of the motif being associated with red-fleshed phenotypes due to the ability of the same MYB10 protein to bind this sequence and enhance its own expression. The two polymorphisms found in this region, alone or in combination, may explain the lack of expression of a470 in the skin.

In this study, we have developed a molecular marker, which can predict the skin color in Japanese plum progenies and could be effectively used in breeding programs. The combination of the haplotypes obtained with the one-primer-pair molecular marker (which can be easily deduced from the allele segregation in the progeny) will identify, in a codominant manner, which seedlings will produce fruits with anthocyanin or non-anthocyanin coloration in the skin. Haplotype data have been shown to be highly informative for MAS (Aranzana et al., 2019), especially when the recombination within interesting regions is low as it occurs in the MYB10 region in *Prunus* LG3. The principal inconvenience of haplotype analysis is that at least two markers usually need to be run, increasing the cost of the analysis. Here, a unique primer can identify most of the haplotypes. Only H4/H5 and H4/H6 combinations amplify the same bands, and an additional primer pair to discern a467 from H6 is required. Given that the six haplotypes observed in this study can predict 91.36% of the phenotypes of the ABL and 83.87% of those in the panel of CV tested, we foresee a high efficiency of this marker in other germplasms. For the remaining accessions (most containing low-frequency alleles), new haplotypes were inferred and need to be confirmed in families; however, color can still be predicted by the presence of a356 and a470 bands.

The color hue and tone have not been studied here. Salazar et al. (2017) found a SNP in “Angeleno” (genotyped here as H1/H3) associated with the purple-skinned phenotypes in progenies, differentiating them from red phenotypes. We identified this SNP in the 5′UTR of a356; the nucleotide associated with higher intensity (G) was observed in a356-H1, while a356-H3 and other alleles not associated with skin color had the alternative nucleotide. In our panel of advanced lines, those with H1 are predominantly purple or black, which is consistent with the results of Salazar et al. (2017). There are many factors involved in coloration hues, such as the anthocyanin molecular structure, pH, copigmentation, temperature, and light (Khoo et al., 2017). Further studies considering anthocyanin levels as well as hue and tonalities are required to identify novel genes and markers for MAS.

## Conclusions

In this study, we have identified high levels of variability in the intronic and intergenic regions of the Japanese plum *MYB10* LG3 cluster, which contains at least three copies of the MYB10.1 gene. In addition to the duplication of this gene, our data are compatible with higher levels of CNV. Methods including long-read sequences would be required to fully characterize the genetic variation in this MYB10 cluster. Fruit color has an important impact on the choice of the consumer, therefore having good molecular markers for MAS is highly desired in breeding. Despite the complexity of the region, using allele cloning, phylogeny, progeny segregation, association test, and gene expression analysis, we succeed in identifying one allele (a356) associated with the skin color. Consequently, markers for this allele can be used with high efficiency for MAS. However, we could not find alleles associated with the flesh color. Here, we also found polymorphisms in the promoter of one of the alternative alleles (a470), which could explain the absence of this pigment in the skin. Further efforts characterizing this region may identify additional polymorphisms involved in fruit coloration.

## Data Availability Statement

The data presented in the study are deposited in the EBI European Nucleotide Archive repository, accession number PRJEB43891.

## Author Contributions

The experiments were conceived and designed by AF and MA. Experiments were conducted by AF. Data was analyzed by AF and MA. WH designed the MYB10F2/MYB10NR2 primers. Bioinformatics analysis was performed by AF, BG-G, FJ-R, and KA. Paper was written by AF and MA. All authors have critically revised the manuscript and approved the final document.

## Conflict of Interest

The authors declare that the research was conducted in the absence of any commercial or financial relationships that could be construed as a potential conflict of interest. The reviewers PM-G and JS declared past co-authorships with one of the authors BG-G and the absence of any ongoing collaboration with any of the authors to the handling editor.
