# Supplementary material for: Characterization of Japanese Plum (Prunus salicina) PsMYB10 Alleles Reveals Structural Variation and Polymorphisms Correlating With Fruit Skin Color
Source: Front Plant Sci. 2021 Jun 8;12:655267. doi: 10.3389/fpls.2021.655267 (PMC8217863; doi:10.3389/fpls.2021.655267)
Supplement: Supplementary file 1 [file Data_Sheet_1.zip › Supplementary Data/SD4. PsMYB10 sequences.docx]

>Prunus salicina MYB10.1 gene fragment, allele a243

GTGTGAGAAAAGGAGCTTGGACTAGAGAGGAAGATGATCTTTTGAGGCAGTGCATTGAGAATCATGGAGAAGGAAAGTGGCACCAAGTTCAAATGATATTACTCGTGATATTACTCGTGAGCCGAGGGGTGCTAAGAGACTTATCCCACATCGGGAATTTTTTGCATTATGCATGGATGCAGGGTTGAACAGGTGCAGGAAGAGCTGTAGACTAAGGTGGTTGAACTATTTGAAGCCAAATATC

>Prunus salicina MYB10.1 gene fragment, allele a350

GTGTGAGAAAAGGAGCTTGGACTAGAGAGGAAGATGATCTTTTGAGGCAGTGCATTGAGAATCATGGAGAAGGAAAGTGGCACCAAGTTCCTAACAAAGCAGGTATGTAAATATAGCTTAGAGAGATATATGATATAGATGGTTAATAAAAGGAAAATGCTAGGAAGAACTTTAGATACCAACTTGTGTACCAACTCTCTTATAGCTCAAATGATATTACTCGTGAGCCGAGGGGTGCTAAGAGACTTATCCCACATCGGGAATTTTTTGCATTATGCATGGATGCAGGGTTGAACAGGTGCAGGAAGAGCTGTAGACTAAGGTGGTTGAACTATTTGAAGCCAAATATC

>Prunus salicina MYB10.1 gene fragment, allele a356

GTGTGAGAAAAGGAGCTTGGACTAGAGAGGAAGATGATCTTTTGAGGCAGTGCATTGAGAATCATGGAGAAGGAAAGTGGCACCAAGTTCCTAACAAAGCAGGTATTAATGTAAATATAGCTTAGAGAGATATATGATATAGATGGTCAATAAAAGGAAAATGCTAGGGAGATCAACTTTAGATACCCACTTGTGTACCAACTCTCTTATAGCTCAAATGATATTACTCATGAGCCGAGGGGTACTAAGAGACTTATCCCACATCGGGAATTTTTTGCATTATGAATGGATGCAGGGTTGAACAGGTGCAGGAAGAGCTGTAGACTAAGGTGGTTGAACTATTTGAAGCCAAATATC

>Prunus salicina MYB10.3 gene fragment, allele a443

GTGTGAGAAAAGGAGCTTGGACTAAAGAGGAAGATGGTCTTCTGATCAAGTGCATGGAGAATCATGGAGAAGGAAAGTGGCACGAGGTTCCTTACAAAGCAGGTATTCATTCATGTAAAATATATATAACTCTCGAAAAGAGGGATATATCTTTAGAAGGTCTTGAACTTCTGCATTCACATGCTCGGCTCAACAGCCACTCGTGCTCCTATATTTTGTTTTTTGTTTTTTGTAGCCAAGCACTTGTGTTTATTTTTTCTCTATCTACATATTTGGCTCAAATGATATCACACGAGAGGCGGACATACTGAGATACGTATTCTACATCGAGAACTACCTCGAGAAAGATGTTATACAATTTTTGTATTATGTATGTATGCAGGCTTAAACAGATGCAGGAAGAGCTGCAGACTACGGTGGTTGAACTATTTGAAGCCAAATATC

>Prunus salicina MYB10.1 gene fragment, allele a454

GTGTGAGAAAAGGAGCTTGGACTAGAGAGGAAGATGATCTTTTGAGGCAGTGCATTGAGAATCATGGAGAAGGAAAGTGGCACCAAGTTCCTAACAAAGCAGGTATTAATGTAAATATAACTCAGAGAGATATATGATATATAGATGGTTTTAATAGGCAGTGAAGCCTTAAATTAGTGATGTTTACAAGGCCTTAAACTTCTCCACTTATTCCCAATTGGTTGCTTTTGATTTTGTGTTTCACTGGCTAGGCCCAATAGTCACTAGTGCTCCCATATTACTCACTATTTATGCTGTCTATGTGTTTAGCTCAAATGATATTGCTCTTGAGCCGAGGGGTGCTAAGAGACTTATCCCACATCGGGAATTTTTTGCATTATGCATGGATGCAGGGTTGAACAGGTGCAGGAAGAGCTGTAGACTAAGGTGGTTGAACTATTTGAAGCCAAATATC

>Prunus salicina MYB10.1 gene fragment, allele a462

GTGTGAGAAAAGGAGCTTGGAGTAGAGAGGAAGATGATCTTTTGAGTCAGTGCATTGAGCATCATGGAGAAGGAAAGTGGCACCAAGTTCCTCACAAAGCAGGTATTAATGAAATATAACTCAGAGAGATATATGATATAGATGGTTAATAAATAGCTAGAGCTTAATTAATAGGCAGTGAAGCCTTAAATTAGTGATGTTTATAAGGCCCTAAACTTCTCCACTTATTGCCAATTGGTTGCTTTTAATTTTGTGTTTCACTGGCTAGGCCCAATAGTCACTAGTGCTCCCATATTACTCACTATTTATGTTGTCTACTTGTTTAGCTCAAATGATATGCTGGTGAGCCGAGGGGTGCTAAGAGACTTATCCCACATCGGGAATTTTTTGCATTATGCATGGATGCACCAGGTGCAGGAAGAGCTGTAGACTAAGGTGGTTGAACTATTTGAAGCCAAATATC

>Prunus salicina MYB10.2 gene fragment, allele a466

GTGTGAGAAAAGGAGCTTGGACTAGAGAGGAAGATGATCTTCTGAGGAAGTGCATTGAGAAACAAGGAGAAGGAAAGTGGCACCAAGTTCCTTACAAAGCAGGTATTAATGTGAAATCTAACTCCAAAAGAGGGATAGATATATGGTATATAGAGCTACAGCTTAATTAAGTACTGAAGCGTTGTCTTAGTATTGTTTCTTAACACACACAAAAACGACATCCTAAACTTCTCCACTCATTGTTAATTGGTTTTCTATACGTGTTCTGCTTAAGGGATGTCATGCATAAAGGCCATTTTGATATGCATGCTGTAACAAAGACAAAAACGAAGTTTAAGACACAGCCTGATAATTTCATGACAAGTTTTGTGATAAAAACTCAATTTTTGCATTATGCACGTGTGCAGGATTAAGCAGATGCAGGAAGAGCTGTAGACTAAGGTGGTTGAACTATTTGAAGCCAAATATC

>Prunus salicina MYB10.1 gene fragment, allele a470

GTGTGAGAAAAGGAGCTTGGACTAGAGAGGAAGATGATCTTTTGAGGCAGTGCATTGAGAATCATGGAGAAGGAAAGTGGCACCAAGTTCCTAACAAAGCAGGTATTAATGTAAATATAGCTCAGAGAGATATATGATATAGATGGTTAATAAAAAGCTAGAGCTTAATTAATAGGCAGTGAAGCCTTAAATTAGTGATGTTTACAAGGCCTTAAACTTCTCCACTTATTCCCAATTGGTTGCTTTTAATTTTGTCTTTCACCGGCCAGGCCCAATAGTCACTAATGCTCCCATATTACTCACTATTTATGTTGTCTACATGTTTAGCCCAAATGATATCACTCGTGAGCCGAGGGGTGCTAAGAGACTTATCCAACATCGGGAATTTTTTGCATTATGCATGGATGCAGGGTTGAACAGGTGCAGGAAGAGCTGTAGACTAAGGTGGTTGAACTATTTGAAGCCAAATATC

>Prunus salicina MYB10.1 gene fragment, allele a473

GTGTGAGAAAAGGAGCTTGGACTAGAGAGGAAGATGATCTTTTGAGGCAGTGCATTGAGAATCATGGAGAAGGAAAGTGGCACCAAGTTCCTAACAAAGCAGGTATTAATGTAAATATAACTCAGATAGATATATGATATGTAGATGGTTAATAAAAAGCTAGAGCTTAATTAATAGGCAGTGAAGCCTTAAATTAGTGATGTTTACAAGGCCTTAAACTTCTCCACTTATTCCCAATTGGTTGCTTTTGATTTTGTGTTTCACTGGCTAGGCCCAATAGTCACTAGTGCTCCCATATTACTCACTATTTATGTTGTCTATGTGTTTAGCTCAAATGATATTGCTCTTGAGCCGAGGGGTGCTAAGAGACTTATCCCACGTCGGGAATTTTTTGCATTATGCATGGATGCAGGGTTGAACAGGTGCAGGAAGAGCTGTAGACTAAGGTGGTTGAACTATTTGAAGCCAAATATC

>Prunus salicina MYB10.3 gene fragment, allele a477

GTGTGAGAAAAGGAGCTTGGACTAAAGAGGAAGATGGTCTTCTGATCAAGTGCATGGAGAATCATGGAGAAGGAAAGTGGCACGAGGTTCCTTACAAAGCAGGTATTCATTCATGTAAAATATATATAACTCTCGAAAAGAGGGATATATAGCTTAATTAAGCAGTGAAGCCTTGCATTAGCGTTGTTTATAAGGTCTTGAACTTCTGCATTCACATGCTCGGCTCAACAGCCACTCGTGCTCCTATATTTTGCTTTTTGTTTTTTGTAGCCAAGCACTTGTGTTTATTTTTTCTCTATCTACATATTTGGCTCAAATGATATCACACGAGAGGCGGACATACTGAGATACATATTCCACATCGAAAACTACCTCAAGAAAGATGTTATACAATGTTTGTATTATGTATGTATGCAGGCTTAAACAGATGCAGGAAGAGCTGCAGACTACGGTGGTTTAACTATTTGAAGCCAAATATC

>Prunus salicina MYB10.3 gene fragment, allele a492

GTGTGAGAAAAGGAGCTTGGACTAAAGAGGAAGATGGTCTTCTGATCAAGTGCATGGAGAATCATGGAGAAGGAAAGTGGCACGAGGTTCCTTACAAAGCAGGTATTCATTCATGTAAAATATATATAACTCTCGAAAAGAGGGATATATATAGATGGTATATATAGCTTAATTAAGCAGTGAAGCCTTGCATTAGCGTTGTTTAGAAGGTCTTGAACTTCTGCATTCACATGCTCGGCTCAACAGCCACTCGTGCTCCTATATTTTGTTTTTTGTTTTTTGTAGCCAAGCACTTGTGTTTATTTTTTCTCTATCTACATATTTGGCTCAAATGATATCACACTAGAGGCGGACATACTGAGATACGTATTCCACATCGAGAACTACCTCGAGAAAGATGTTATACAATTTTTGTATTATGTATGTATGCAGGCTTAAACAGATGCAGGAAGAGCTGCAGACTACGGTGGTTGAACTATTTGAAGCCAAATATC

>Prunus salicina MYB10.3 gene fragment, allele a495

GTGTGAGAAAAGGAGCTTGGACTAAAGAGGAAGATGGTCTTCTGATCAAGTGCATGGAGAATCATGGAGAAGGAAAGTGGCACGAGGTTCCTTACAAAGCAGGTATTCATTCATGTAAAATATATATAACTCTCGAAAAGAGGGATATATATAGATGGTATATATATAGCTTAATTAAGCAGTGAAGCCCTGCATTAGCGTTGTTTAGAAGGTCTTGAACTTCTGCATTCACATGCTCGGCTCAACAGCCACTCGTGCTCCTATATTTTGTTTTTTGTTCTTTGTAGCCAAGCACTTGTGTTTATTTTTTCTCTATCTACATATTTGGCTCAAATGATATCACACGAGAGGCGGACATACTGAGATACGTATTCTACATCGAGAACTACCTCGAGAAAGATGTTATACAATTTTTGTATTATGTATGTATGCAGGCTTAAACAGATGCAGGAAGAGCTGCAGACTACGGTGGTTGAACTATTTGAAGCCAAATATC

>Prunus salicina MYB10.1A gene, allele a356 from haplotype H1

ATGGAGGGCTCTAACCTGGGTGTGAGAAAAGGAGCTTGGACTAGAGAGGAAGATGATCTTTTGAGGCAGTGCATTGAGAATCATGGAGAAGGAAAGTGGCACCAAGTTCCTAACAAAGCAGGTATTAATGTAAATATAGCTTAGAGAGATATATGATATAGATGGTCAATAAAAGGAAAATGCTAGGGAGATCAACTTTAGATACCCACTTGTGTACCAACTCTCTTATAGCTCAAATGATATTACTCATGAGCCGAGGGGTACTAAGAGACTTATCCCACATCGGGAATTTTTTGCATTATGAATGGATGCAGGGTTGAACAGGTGCAGGAAGAGCTGTAGACTAAGGTGGTTGAACTATTTGAAGCCAAATATCAAGAGAGGAGACTTCACGGAAGATGAAGTAGATCTAATAACTAGGCTTCACAAGCTTTTAGGAAACAGGTACCATTAAATGTCTCTTTCTTTATCCCACATCGTACTTTCATCACATAACATTAAAAAAAAAAAAACTAAAATCCACAATCGCCGACATSMATCCCGTGCTTTGTTTTCTAATATTATATCTTCTGTTTCTCAGTAAGCGTGCACAAGCACAAAAAGCACTAGAAGGGCCCATGTAGCCATGCATGATGTATCTTAGTCTCTGTGAATCGTAAAACATAGTGATCATATATGTTAGAACATTCACAGAAGATTTTTTTTTATCACAAATAGATGAGGTTTGCGAAATCATCTACTTTTTTTGTGTGTGTATCGTAGCACAAATGTATTGAATATTGATTTATTTTCTGATGCTATCTATCGAAGGTGGTCATTGATTGCTGGAAGGCTTCCAGGAAGGACAGCGAATGATGTGAAAAATTATTGGAACACTCGACTGCGGATGGATTCTCGCCTGAAAAAGGTGAAAGATAAACCCCAAGAAACAATAAAGGCCATCGTAATAAGACCTCAACCCCGAAGCTTCTTAAAGAGTTCAAATTGTTTGAGCAGTAAAGAACCAATTTTGGACCATATTCAAACAGTCGAGAATTTTAGTACGCCGTCACAATCATCACCATCAACAAAAAACGGAAAGGATTGGTGGGAAACCTTTTTAGATGACGAGGATGTTTTTGAAAAAGCTACATGCTATGGTCTAGCGTTAGAGGAAGGAGAGTTCACAAGTTTTTGGGTTGATGATATGCCACAATCGAAAAGACAGTGTACCAATGTTACAGAAGAAGGACTAGGTACAGGTGATTTCTCTTTTAACGTGGACTTTTGGAATCATTAA

>Prunus salicina MYB10.1A gene, allele a356 from haplotype H3

ATGGAGGGCTCTAACTTGGGTGTGAGAAAAGGAGCTTGGACTAGAGAGGAAGATGATCTTTTGAGGCAGTGCATTGAGAATCATGGAGAAGGAAAGTGGCACCAAGTTCCTAACAAAGCAGGTATTAATGTAAATATAGCTTAGAGAGATATATGATATAGATGGTTAATAAAAGGAAAATTCTAGGGAGATCAACTTTAGATACCAACTTGTGTACAAACTCTCTTACAGCTCAAATGATATTACTCGTGAGCCGAGGGGTGCTAAGAGACTTATCCCACATCGGGAATTTTTTGCATTATGCATGGATGCAGGGTTGAACAGGTGCAGGAAGAGCTGTAGACTAAGGTGGTTGAACTATTTGAAGCCAAATATCAAGAGAGGAGACTTCACGGAAGATGAAGTAGATCTAATAATTAGGCTTCACAAGCTTTTAGGAAACAGGTACCAATAAATGTCTCTTTCTTTATCCCACATCGTTCTTTCATCACATAACATTAAAAAATAAAAAAAATAAAAACTAAAATCCACAATCGCCGACATGCATCCCGTGCTTTGTTTTCTAATATTATATCTTCTGTTTCTCAGTAAGCGTGCACAAGCACAAAAAGCACTAGAAGGGCCATGTAGCCATGCATGATGTACCTTAGTCTCTGTGAAATCGTAAAACATAGTGATCATATATGTTAGAACATTTACAGAAGGTTTTTTTTTTATCAGAAATAGTTCTGAGGTTTGTGAAATTATCGTATTTCGTCTTTAATTAATTTATTATTATTATTATTATTATTTTTTGTGATACTAATGGTCCTTAAGGTTATCATTCACACATCAAAATGATCTTTGTCATCAGTTTCCGTCAAATTTTCTGTTAAAATGCTGATGTGGCATATATGTGGAGCCACACATATAACAATATAGTGCCACGTAGCTTTAATAAAAGATTTAAATCCCGAGCCGGTTCTTTGCTTGCCTGACCATCACCCTCAAATCTGATGTGAGAGAGAGAGAGAGAGAGAGAGAGAGAGAGAGAGAGGGGGGGTGCCGGGTTTGAAAGAAGAGGGAGAGAGAGATTTTTTTATATTATATTTTTTTAAAGTTTGAATCTTATCTTTTTTAAATATATAAATATTTTATTTAAACCCACGTGGCTATAAAATAGTGGGTGTGTGGCCCACATATATGCCATATCATCTTTTTAAAATTTGACAAAAGTTGCCACATATATGCCATATCCTCTTTTTAAAATTTGACAAAAGTTGACGGCGTGGTTTGATTTATTTTAACTGGTGTTATTTCCACCCACTTTGCTTATTTTCCCACTCTTTTTATTTTCAAATTTTTAAAAACATTTTTCTCCCTTTACAAAGTGACTTTTAGGGACAAATTTAAAATCTCTATTTATTAATAAAAGCTTGACACATCATTAAAATTCACTCTGGGGGTAAAAAAAAANNNNNNNNNNNNNNNNNNNNNNNNNNNNNNNNNNNNNNNNNNNNNNNNNNNNNNNNNNNNNNNNNNNNNNNNNNNNNNNNNNNNNNNNNNNNNNNNNNNNNNNNNNNNNNNNNNNNNNNNNNNNNNNNNNNNNNNNNNNNNNNNNNNNNNNNNNNNNNNNNNNNNNNNNNNNNNNNNNNNNNNNNNNNNNNNNNNNNNNNNNNNNNNNNNNNNNNNNNNNNNNNNNNNNNNNNNNNNNNNNNNNNNNNNNAATCTTGTGTGAGAGAGATAAATGAAAATAAAATAAAAATAATATTTTATTTTTTATATCTCAGAGAGAGAGAGAGAGAGAGAGAGAGAGAGAGAGAGAGAGAAGCAAAGGAACCATTCCATGATCATGGGAATCAAAATACAAAGGGTACAATGGGTTCTATTTGGGTTTTGAGGGTTTATATTTCTAAGAAGAGAGGTTGGGTCGCAAGGAGGAGGGAGAGACTTAAGCGGGGATGGGCTAATTCAAGAATTTTTTAATTGTAATGGAAAATATGAAAATGCAATTTTTTTAAAAAAAAATTACTAATGATTAGCAAAAAGAATTTACTAGAAGTAATAATTAAAGGGTATTTTTAGGAATAATGGTGGGTGGAAAGATAAGTTCGTGTTCTTTCAAATTTACATGGTGGGTGAGAAAATAAGACGAGTGAGAAAATAAGACGAGGGGGTAGAAATAACAGCATTTCTACTTTTTTTGTGTGTGTATCATAGCACAAATGTATTAATTTATTTTCTAATGCTATCTGTCGAAGGTGGTCATTGATTGCTGGAAGGCTTCCAGGAAGGACAGCGAATGATGTGAAAAATTATTGGAACACTCGACTGCGGATGGTTTCTCGCCTGAAAAAGGTGAAAGATAAACCCCAAGAAACAATAAATGCCATCGTAATAAGACCTCAACCCCGAAGTTTCATCAAGAGTTCAAATTGTTTGAGCAGTAAAGAACCAATTTTGGACCATATTCAAACAGTCGAGAATTTTAGTACGCCGTCACAATCATCACCATCAACAAAGAACGGAAATGATTGGTGGGAAACCTTTTTAGATGACGAGGATGTTTTTGAAAAAGCTACATGCTATGGTCTAGCTTTAGATGAAGAAGAGTTCACAAGTTTTTGGGTTGATGATATGCCACAATCGAAAAGACAGTGTACCAATGTTACAGAAGAAGGACTAGGTACAGGTGATTTCTCTTTTAACGTGGACTTTTGGAATCATTAA

>Prunus salicina MYB10.1A gene, allele a470 from haplotype H2

ATGGAGGGCTCTAACTTGGGTGTGAGAAAAGGAGCTTGGACTAGAGAGGAAGATGATCTTTTGAGGCAGTGCATTGAGAATCATGGAGAAGGAAAGTGGCACCAAGTTCCTAACAAAGCAGGTATTAATGTAAATATAGCTCAGAGAGATATATGATATAGATGGTTAATAAAAAGCTAGAGCTTAATTAATAGGCAGTGAAGCCTTAAATTAGTGATGTTTACAAGGCCTTAAACTTCTCCACTTATTCCCAATTGGTTGCTTTTAATTTTGTCTTTCACCGGCCAGGCCCAATAGTCACTAATGCTCCCATATTACTCACTATTTATGTTGTCTACATGTTTAGCTCAAATGATATCACTCGTGAGCCGAGGGGTGCTAAGAGACTTATCCAACATCGGGAATTTTTTGCATTATGCATGGATGCAGGGTTGAACAGGTGCAGGAAGAGCTGTAGACTAAGGTGGTTGAACTATTTGAAGCCAAATATCAAGAGAGGAGACTTTACGGAAGATGAAGTAGATCTAATAATTAGGCTTCACAAGCTTTTAGGAAACAGGTACCAATATGTGTCTCTTTCCTTATCCCACATGGTTCTTTCATCACATACCAAATCCACAATCGCCGACATGCATCCCGTGCTTTGTTTTCTAATATTATATCTTCTGTTTCTCAGTAAGCGTGCACAAGCACAAAAAGCACTGGAAGGGCCATGTAGCCATGCATGATGTATCTTAGTCTCTGTGAATCGTAAAACATAGTGATCATATATGTTAGAACATTTACAGAAGGTGGTCATTGATTTATTTTCTGATGCTATCTGTCGAAGGTGGTCATTGATTGCTGGAAGGCTTCCAGGAAGGACAGCGAATGATGTGAAAAATTATTGGAACACTCGACTGCGGATGGATTCTCGCCTGAAAAAGGTGAAAGATAAACCCCAAGAAACAATAAAGGCCATCGTAATAAGACCTCAACCCCGAAGCTTCATCAAGAGTTCAAGTTGTTTGAGCAGTAAAGGACCAATTTTGGACCATATTCAAACAGTCGAGAATTTTAGTACGCCGTCACAATCATCACCATCAACAAAGAACGGAAATGATTGGTGGGAAACCTTTTTAGATGACGAGGATGTTTTTGAAAAAGCTACATGCTATGGTCTAGCGTTAGAGGAAGAAGAGTTCACAAGTTTTTGGGTTGATGATATGCCACAATCGAAAAGACAGTGTACCAATGTTACAGAAGAAGGACTAGGTACAGGTGATTTCTCTTTTAACGTGGACTTTTGGAATCATTAA

>Prunus salicina MYB10.1A gene, allele a470 from haplotype H4

ATGGAGGGCTCTAACTTGGGTGTGAGAAAAGGAGCTTGGACTAGAGAGGAAGATGATCTTTTGAGGCAGTGCATTGAGAATCATGGAGAAGGAAAGTGGCACCAAGTTCCTAACAAAGCAGGTATTAATGTAAATATAGCTCAGAGAGATATATGATATAGATGGTTAATAAAAAGCTAGAGCTTAATTAATAGGCAGTGAAGCCTTAAATTAGTGATGTTTACAAGGCCTTAAACTTCTCCACTTATTCCCAATTGGTTGCTTTTAATTTTGTCTTTCACCGGCCAGGCCCAATAGTCACTAATGCTCCCATATTACTCACTATTTATGTTGTCTACATGTTTAGCTCAAATGATATCACTCGTGAGCCGAGGGGTGCTAAGAGACTTATCCAACATCGGGAATTTTTTGCATTATGCATGGATGCAGGGTTGAACAGGTGCAGGAAGAGCTGTAGACTAAGGTGGTTGAACTATTTGAAGCCAAATATCAAGAGAGGAGACTTTACGGAAGATGAAGTAGATCTAATAATTAGGCTTCACAAGCTTTTAGGAAACAGGTACCAATATGTGTCTCTTTCCTTATCCCACATGGTTCTTTCATCACATACCAAATCCACAATCGCCGACATGCATCCCGTGCTTTGTTTTCTAATATTATATCTTCTGTTTCTCAGTAAGCGTGCACAAGCACAAAAAGCACTGGAAGGGCCATGTAGCCATGCATGATGTATCTTAGTCTCTGTGAATCGTAAAACATAGTGATCATATATGTTAGAACATTTACAGAAGGTGGTCATTGATTTATTTTCTGATGCTATCTGTCGAAGGTGGTCATTGATTGCTGGAAGGCTTCCAGGAAGGACAGCGAATGATGTGAAAAATTATTGGAACACTCGACTGCGGATGGATTCTCGCCTGAAAAAGGTGAAAGATAAACCCCAAGAAACAATAAAGGCCATCGTAATAAGACCTCAACCCCGAAGCTTCATCAAGAGTTCAAGTTGTTTGAGCAGTAAAGGACCAATTTTGGACCATATTCAAACAGTCGAGAATTTTAGTACGCCGTCACAATCATCACCATCAACAAAGAACGGAAATGATTGGTGGGAAACCTTTTTAGATGACGAGGATGTTTTTGAAAAAGCTACATGCTATGGTCTAGCGTTAGAGGAAGAAGAGTTCACAAGTTTTTGGGTTGATGATATGCCACAATCGAAAAGACAGTGTACCAATGTTACAGAAGAAGGACTAGGTACAGGTGATTTCTCTTTTAACGTGGACTTTTGGAATCATTAA

>Prunus salicina MYB10.1 gene, allele a467 from haplotype H6

ATGGAGGGCTCTAACTTGGGTGTGAGAAAAGGAGCTTGGACTAGAGAGGAAGATGATCTTTTGAGGCAGTGCATTGAGAATCATGGAGAAGGAAAGTGGCACCAAGTTCCTAACAAAGCAGGTATTAATGTAAATATAGCTCAGAGAGATATATGATATAGATGGTTAATAAAAAGCTAGAGCTTAATTAATAGGCAGTGAAGCCTTAAATTAGTGATGTTTACAAGGCCTTAAACTTCTCCACTTATTCCCAATTGGTTGCTTTTATTTTTGTCTTTCACCGGCCAGGCCCAATAGTCACTAATGCTCCCATATTACTCACTATTTATGTTGTCTACATGTTTAGCTCAAATGATATCACTCGTGAGCCGAGGGGTGCTAAGAGACTTATCCAACATAATTTTTTGCATTATGCATGGATGCAGGGTTGAACAGGTGCAGGAAGAGCTGTAGACTAAGGTGGTTGAACTATTTGAAGCCAAATATCAAGAGAGGAGACTTTGCGGAAGATGAAATAGATCTAATAATTAGGCTTCACAAGCTTTTAGGAAACAGGTACCAATAAATGTCTCTTTCCTTATCCCACATGGTTCTTTCATCACATACCAAATCCACAATCGCCGACATGCATCCCGTGCTTTGTTTTCTAATATTATATCTTCTGTTTCTCAGTAAGCGTGCACAAGCACAAAAAGCACTGGAAGGGCCATGTAGCCATGCATGATGTATCTTAGTCTCTGTGAATCGTAAAACATAGTGATCATATATGTTAGAACATTTACAGAAGGTGGTCATTGATTTATTTTCTGATGCTATCTGTCGAAGGTGGTCATTGATTGCTGGAAGGCTTCCAGGAAGGACAGCGAATGATGTGAAAAATTATTGGAACACTCGACTGCGGATGGATTCTCGCCTGAAAAAGGTGAAAGATAAACCCCAAGAAACAATAAAGGCCATCGTAATAAGACCTCAACCCCGAAGCTTCATCAAGAGTTCAAATTGTTTGAGCAGTAAAGAACCAATTTTGGACCATATTCAAACAGTCGAGAATTTTAGTACGCCGTCACAATCATCACCATCAACAAAGAACGGAAATGATTGGTGGGAAACCTTTTTAGATGACGAGGATGTTTTTGAAAAAGCTACATGCTATGGTCTAGCGTTAGAGGAAGAAGAGTTCACAAGTTTTTGGGTTGATGATATGCCACAATCGAAAAGACAGTGTACCAATGTTACAGAAGAAGGACTAGGTACAGGTGATTTCTCTTTTAACGTGGACTTTTGGAATCATAA

>Prunus salicina MYB10.2 gene CDS, allele a466

ATGGAGGGTTATAACTTGGGTGTGAGAAAAGGAGCTTGGACTAGAGAGGAAGATGATCTTCTGAGGCAGTGCATTGAGAAACAAGGAGAAGGAAAGTGGCACCAAGTTCCTTACAAAGCAGGATTAAGCAGATGCAGGAAGAGCTGTAGACTAAGGTGGTTGAACTATTTGAAGCCAAATATCAAGAGAGGAGACTTTATGGAAGATGAAGTAGATCTAATAATTAGGCTTCACAAGCTTTTAGGAAACAGGTGGTCATTGATTGCTCGAAGACTTCCGGGAAGGACTGCCAATGATGTGAAAAATTACTGGAACACCCGATTGCGGACGGATTATTGCATGAAAAAGATGAAAGACAAATCCCAAGAAACAATAAAGACCATAATAAGGCCACAACCAAGAAGATTCACCAAAAGTTCAAATTGTTTGAGTTTTAAAGAACCAATTTTGGACCATACTCAACTAGAAGAGAATTTTAGTACGACATCACAAACATCAACATCAACAAGGATTGGAAGTGATTGGTGGGAGACCTTTTTAGATGACAAGGATGCTACTGAAACAGCTACAGGTTCTGGTCTTGGGTTAGATGAAGAACTGCTCGCAAGTTTTTGGGTTGATGATAATATGCCACAATCGACAAGAACATGCGTCAATTTTTCTGAGGAAGGATTAAGTAGAGGTGATTTCTCTTTTAGCGTGGACCTTTGGAATCATTCAAATGAAGAATAG

>Prunus salicina MYB10.3 gene CDS, allele a492

ATGGGGGGAAATAACTTGGATGTGAGAAAAGGAGCTTGGACTAAAGAGGAAGATGGTCTTCTGATCAAGTGCATGGAGAATCATGGAGAAGGAAAGTGGCACGAGGTTCCTTACAAAGCAGGCTTAAACAGATGCAGGAAGAGCTGCAGACTACGGTGGTTGAACTATTTGAAGCCAAATATCAAGAGAGGAGAGTTTACAGAGGATGAAGTTGATCTAATAATTAGGCTTCACAAGCTTTTAGGAAACAGGTGGTCATTGATTGCTGGAAGACTTCCAGGAAGGACATCGAACGGTGTGAAAAATTATTGGCACACCCGACTGCGGACAAATTCTCTCGTGAAAAGGACTACGAAAAATAAATTCCAAGAAACAATAAGGCCTCAACCGCGAAGTTTCACCAAAAGTTCAAATTGTTTGAATTTTAAAGAACCAATTTTGGACCATACTCAACTAGAAAAGAATTTTAGTACGCCATCATAAACATCAACATCAACATCAACAAGGATTGGAAGTGATTGGTGGGAGACCTTTTTAGATGACAAGGATGATGCTACTGAAAGAGCTACAGGTTCTGGTCTCGGGTTAGATGAAGAACTGCTCGGAAGTTTTTGGGTTGATGATGATATGCCACAATCGGCAAGAACATGCATCAATTTTTCTGAAGAAGAACTGAGTATAAGTGATTTCGCTTTTAACTTGGACCTTGGAATCATCAAAAGATGA

>Prunus salicina MYB10.1A gene cDNA, allele a356 from haplotype H1

ATGGAGGGCTCTAACTTGGGTGTGAGAAAAGGAGCTTGGACTAGAGAGGAAGATGATCTTTTGAGGCAGTGCATTGAGAATCATGGAGAAGGAAAGTGGCACCAAGTTCCTAACAAAGCAGGGTTGAACAGGTGCAGGAAGAGCTGTAGACTAAGGTGGTTGAACTATTTGAAGCCAAATATCAAGAGAGGAGACTTCACGGAAGATGAAGTAGATCTAATAACTAGGCTTCACAAGCTTTTAGGAAACAGGTGGTCATTGATTGCTGGAAGGCTTCCAGGAAGGACAGCGAATGATGTGAAAAATTATTGGAACACTCGACTGCGGATGGATTCTCGCCTGAAAAAGGTGAAAGATAAACCCCAAGAAACAATAAAGGCCATCGTAATAAGACCTCAACCCCGAAGCTTCTTAAAGAGTTCAAATTGTTTGAGCAGTAAAGAACCAATTTTGGACCATATTCAAACAGTCGAGAATTTTAGTACGCCGTCACAATCATCACCATCAACAAAAAACGGAAAGGATTGGTGGGAAACCTTTTTAGATGACGAGGATGTTTTTGAAAAAGCTACATGCTATGGTCTAGCGTTAGAGGAAGAAGAGTTCACAAGTTTTTGGGTTGATGATATGCCACAATCGAAAAGACAGTGTACCAATGTTACAGAAGAAGGACTAGGTACAGGTGATTTCTCTTTTAACGTGGACTTTTGGAATCATTAA
