# Supplementary material for: Characterization of Japanese Plum (Prunus salicina) PsMYB10 Alleles Reveals Structural Variation and Polymorphisms Correlating With Fruit Skin Color
Source: Front Plant Sci. 2021 Jun 8;12:655267. doi: 10.3389/fpls.2021.655267 (PMC8217863; doi:10.3389/fpls.2021.655267)
Supplement: Supplementary file 1 [file Data_Sheet_1.zip › Supplementary Tables/ST1. SSR markers used to construct LG3 genetic map of P1, P2 and P3 families.docx]

Supplementary Table ST1. SSR markers used to contruct LG3-*PsMYB10* genetic maps of P1, P2 and P3 families.

| **SSR marker** | **Families genotyped** | **Position in Peach Genome v2.0** | **Reference** |
| --- | --- | --- | --- |
| **MA039a** | P1, P2 and P3 | Pp03: 17772071-17772260 | Yamamoto *et al*., 2002 |
| **UDAp-496** |  | Pp03: 16591013-16591165 | Messina *et al*., 2004 |
| **PaCITA10** |  | Pp03: 19499241-19499378 | Lopes *et al*., 2002 |
| **EPPCU0532** |  | Pp03: 26704358-26704530 | Howad *et al*., 2005 |
| **BPPCT007** | P1 | Pp03: 3602939-3603083 | Dirlewanger *et al*., 2002 |
| **BPPCT039** | P2 and P3 | Pp03: 6662508-6662632 | Dirlewanger *et al*., 2002 |
