# Supplementary material for: Characterization of Japanese Plum (Prunus salicina) PsMYB10 Alleles Reveals Structural Variation and Polymorphisms Correlating With Fruit Skin Color
Source: Front Plant Sci. 2021 Jun 8;12:655267. doi: 10.3389/fpls.2021.655267 (PMC8217863; doi:10.3389/fpls.2021.655267)
Supplement: Supplementary file 1 [file Data_Sheet_1.zip › Supplementary Tables/ST2. Designed primers used in Japanese plum.docx]

Supplementary Table ST2. Primer combinations used to amplify *PsMYB10* sequences

| **Marker name** | **Forward/reverse** | **Sequence** | **Ta (ºC)** | **Product range (bp)** | **Details** |
| --- | --- | --- | --- | --- | --- |
| **MYB10F2** | For | GTGTGAGAAAAGGAGCTT | 55 | 243-500 | MYB10 marker |
| **MYB10NR2** | Rev | GATATTTGGCTTCAAATAGTTC |  |  |  |
| **M101_f** | For | CTGTTATCAAGCCCTACATG | 57 |  | MYB10.1-a356 and MYB10.1-a470 full gene amplification |
| **M101_r** | Rev | GGTCTTTTGACAGCCG |  |  |  |
| **M102_f** | For | CTGGCTGCAAGCATAC | 57 |  | MYB10.2 full gene amplification |
| **M102_r** | Rev | GTGGGACAAACACTCTC |  |  |  |
| **M103_f** | For | ATAGGAACTAGCAGGCAC | 57 |  | MYB10.3 full gene amplification |
| **M103_r** | Rev | AGTTGCTAATAATTGCTACTAGG |  |  |  |
| **Pav3_9371** | For | GAGTCGAGCTTGGGTC | 54 |  | Upstream amplification of MYB10.1-a356 (1R), a470 (2R) or a243 (5R) |
| **MYB10_int1_1R** | Rev | AGAGTTGGTACACAAGTGGGTATC | 54 |  |  |
| **MYB10_int1_2R** | Rev | AGCACTAGTGACTATTGGGCC | 54 |  |  |
| **MYB10_int1_5R** | Rev | CCTCGGCTCACGAGTAATATCACGAG | 54 |  |  |
| **M101_RT_F** | For | TGGACACGAGACATTGCACG | 60 | 950 bp | Genomic and cDNA MYB10.1 amplification |
| **M101_RT_R** | Rev | CAATGGTCTTTTGACAGCCGC | 60 |  |  |
| **MYB10NR4** | Rev | TTCCTGCACCTGTTCAAC | 55 |  | Used with MYB10F2 to discern a467 |
