# Supplementary material for: Characterization of Japanese Plum (Prunus salicina) PsMYB10 Alleles Reveals Structural Variation and Polymorphisms Correlating With Fruit Skin Color
Source: Front Plant Sci. 2021 Jun 8;12:655267. doi: 10.3389/fpls.2021.655267 (PMC8217863; doi:10.3389/fpls.2021.655267)
Supplement: Supplementary file 1 [file Data_Sheet_1.zip › Supplementary Tables/ST3. BLAST results for the cloned alleles.docx]

Supplementary Table ST3. Sequence homology of cloned sequences with *Prunus* LG3-*PpMYB10* sequences

| Fragment allele | Homologous gene from *Prunus persica* genome V2.0 | NCBI blastn | | |
| --- | --- | --- | --- | --- |
|  |  | R2R3 MYB TF match | Identity (%) | Query coverage (%) |
| a243 | Prupe.3G163100  (*PpMYB10.1*) | *Prunus domestica subsp. insititia* R2R3 MYB transcription factor 10 gene, complete cds  (EU153579.1) | 97.84 | 93 |
| a350 |  |  | 97.97 | 85 |
| a356 |  |  | 96.82 | 84 |
| a454 |  |  | 95.36 | 100 |
| a462 |  |  | 94.51 | 100 |
| a470 |  |  | 96.62 | 100 |
| a473 |  |  | 99.16 | 100 |
| a466 | Prupe.3G163000  (*PpMYB10.2*) | *Prunus domestica* R2R3 MYB transcription factor 10 gene, complete cds  (EU153580.1) | 98.93 | 100 |
| a443 | Prupe.3G163300  (*PpMYB10.3*) | *Prunus avium* R2R3-MYB transcription factor (*MYBA1*) gene, complete cds  (GU938680.1) | 90.65 | 43 |
| a477 |  |  | 90.65 | 22 |
| a492 |  |  | 79.49 | 62 |
| a495 |  |  | 79.57 | 62 |
