# Supplementary material for: Characterization of Japanese Plum (Prunus salicina) PsMYB10 Alleles Reveals Structural Variation and Polymorphisms Correlating With Fruit Skin Color
Source: Front Plant Sci. 2021 Jun 8;12:655267. doi: 10.3389/fpls.2021.655267 (PMC8217863; doi:10.3389/fpls.2021.655267)
Supplement: Supplementary file 1 [file Data_Sheet_1.zip › Supplementary Tables/ST4. Illumina alignments results.docx]

|  |  | **Genome** | **LG3** | **MYB10  region** | **MYB10  genic region** | **MYB10  intergenic region** |
| --- | --- | --- | --- | --- | --- | --- |
|  |  | ***P. persica*** | | | | |
| **depth** | **C20** | 22 | 23 | 24 | 40 | 17 |
|  | **C46** | 74 | 73 | 63 | 135 | 36 |
| **coverage** | **C20** | 0,74 | 0,72 | 0,34 | 0,95 | 0,26 |
|  | **C46** | 0,78 | 0,77 | 0,37 | 0,97 | 0,3 |
|  |  | ***P. dulcis*** | | | | |
| **depth** | **C20** | 22 | 22 | 28 | 30 | 27 |
|  | **C46** | 78 | 78 | 85 | 101 | 66 |
| **coverage** | **C20** | 0,64 | 0,63 | 0,74 | 0,93 | 0,65 |
|  | **C46** | 0,84 | 0,84 | 0,79 | 0,98 | 0,7 |
|  |  | ***P. avium*** | | | | |
| **depth** | **C20** | 23 | 24 | 13 | 11 | 14 |
|  | **C46** | 73 | 77 | 30 | 21 | 33 |
| **coverage** | **C20** | 0,77 | 0,79 | 0,47 | 0,47 | 0,47 |
|  | **C46** | 0,81 | 0,83 | 0,54 | 0,53 | 0,54 |

Supplementary Table ST4. Results of Illumina data aligned against three *Prunus* genomes
