# Supplementary material for: Characterization of Japanese Plum (Prunus salicina) PsMYB10 Alleles Reveals Structural Variation and Polymorphisms Correlating With Fruit Skin Color
Source: Front Plant Sci. 2021 Jun 8;12:655267. doi: 10.3389/fpls.2021.655267 (PMC8217863; doi:10.3389/fpls.2021.655267)
Supplement: Supplementary file 1 [file Data_Sheet_1.zip › Supplementary Tables/ST5. Chi-square test of the MYB10 amplified alleles in the parental collection.docx]

Supplementary Table ST5. Results of χ^2^_(1df)_ test in the panel of 81 accessions. The presence of allele a356 is statistically associated (p=1,96×10^-18)^ with the observation of anthocyanin-based color in the fruit skin.

| **Allele name** | **Allele frequency** | **Chi-square p-value** | |
| --- | --- | --- | --- |
|  |  | **Skin color** | **Flesh color** |
| a243 | 0,05 | 5,06×10^-1^ | 1,06×10^-1^ |
| a350 | 0,42 | 2,64×10^-8^ | 6,47×10^-1^ |
| a356 | 0,64 | 1,96×10^-18^ | 3,65×10^-1^ |
| a443 | 0,31 | 2,21×10^-6^ | 2,38×10^-2^ |
| a454 | 0,44 | 7,42×10^-8^ | 9,19×10^-1^ |
| a462 | 0,54 | 7,55×10^-2^ | 1,70×10^-3^ |
| a470 | 0,77 | 2,14×10^-3^ | 7,16×10^-4^ |
| a473 | 0,27 | 5,22×10^-4^ | 2,31×10^-2^ |
| a477 | 0,19 | 7,55×10^-1^ | 2,23×10^-1^ |
| a492 | 0,73 | 2,08×10^-1^ | 1,37×10^-4^ |
| a495 | 0,22 | 2,93×10^-4^ | 2,12×10^-2^ |
