# Supplementary figures and images for: Characterization of Japanese Plum (Prunus salicina) PsMYB10 Alleles Reveals Structural Variation and Polymorphisms Correlating With Fruit Skin Color
Source: Front Plant Sci. 2021 Jun 8;12:655267. doi: 10.3389/fpls.2021.655267 (PMC8217863; doi:10.3389/fpls.2021.655267)

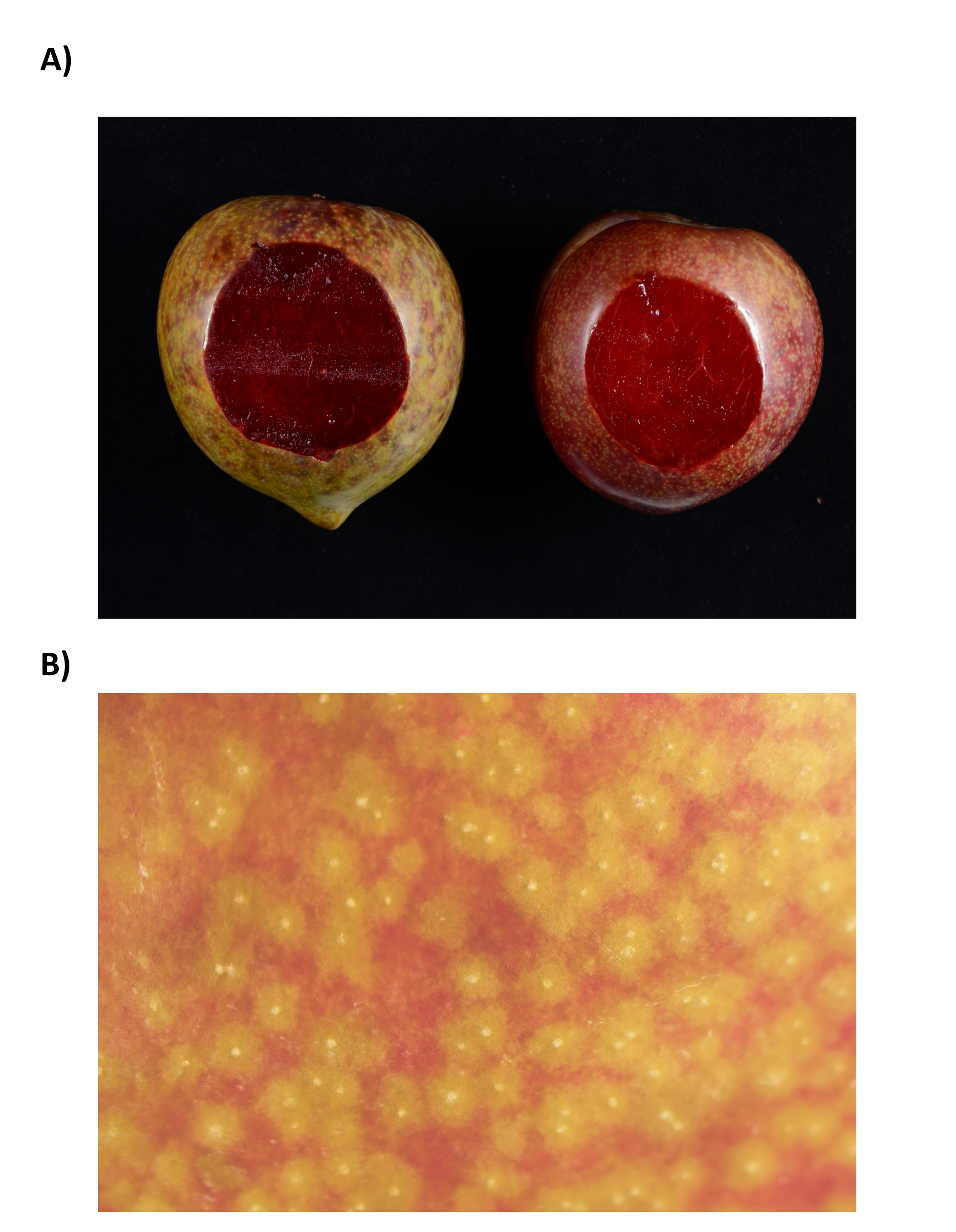

Supplement: Supplementary file 1 [file Data_Sheet_1.zip › Supplementary Figures/SF1. (A) Fruits with mottled skin phenotype; (B) Detail of the skin speckles.tif]

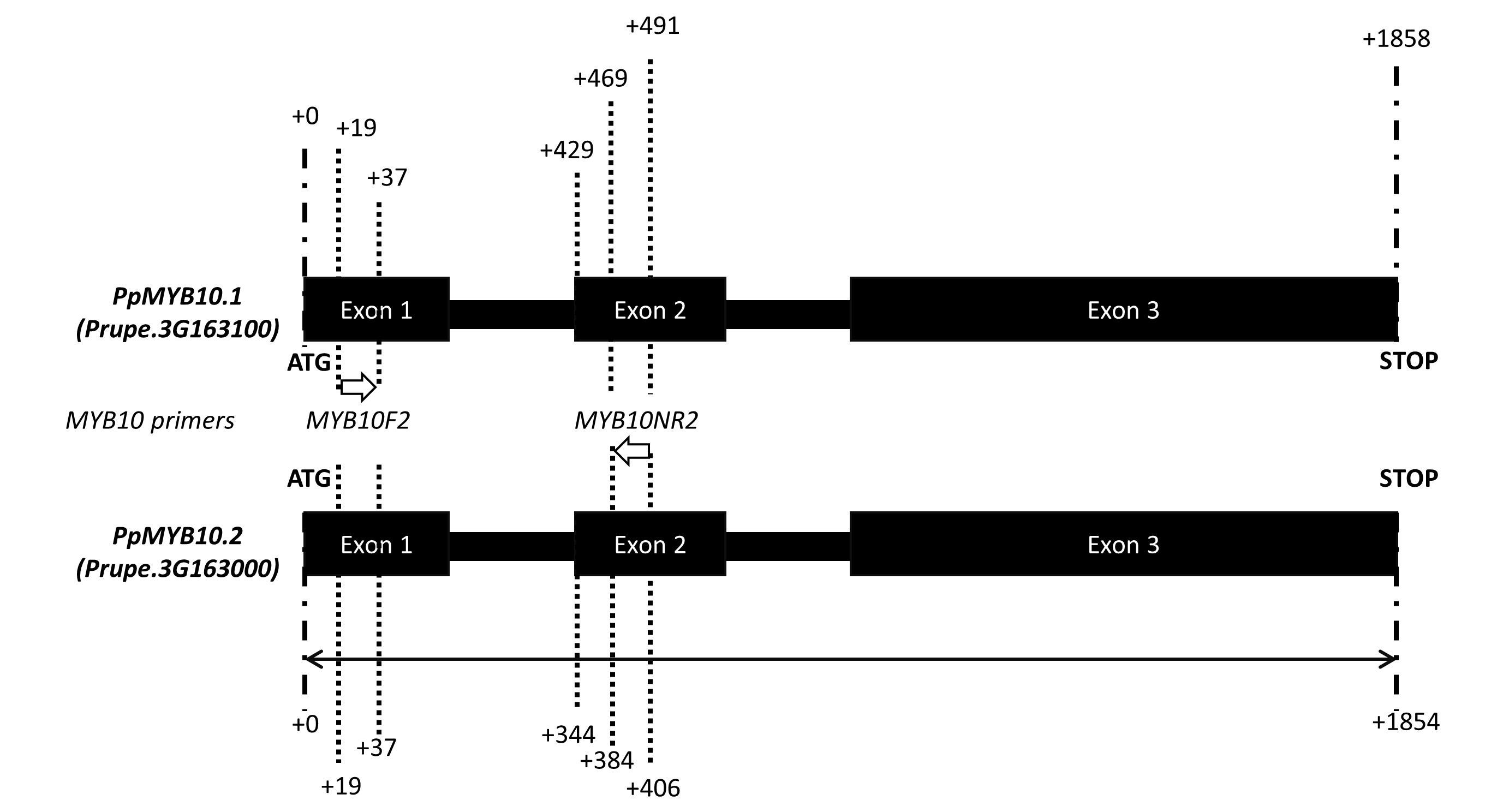

Supplement: Supplementary file 1 [file Data_Sheet_1.zip › Supplementary Figures/SF2. Primer positions on the PpMYB10.1 and PpMYB10.2 genes.tif]

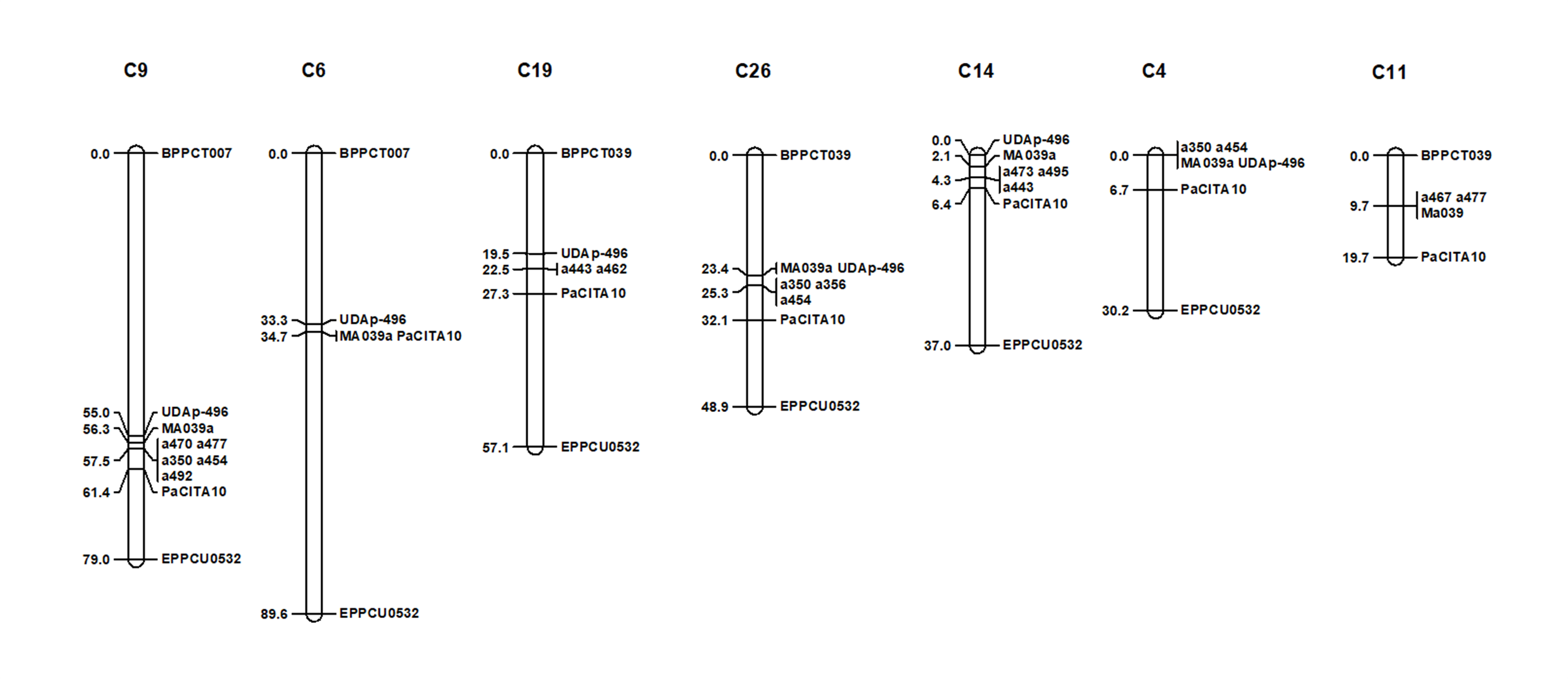

Supplement: Supplementary file 1 [file Data_Sheet_1.zip › Supplementary Figures/SF3. Genetic map of the Japanese plum MYB10 alleles.tif]

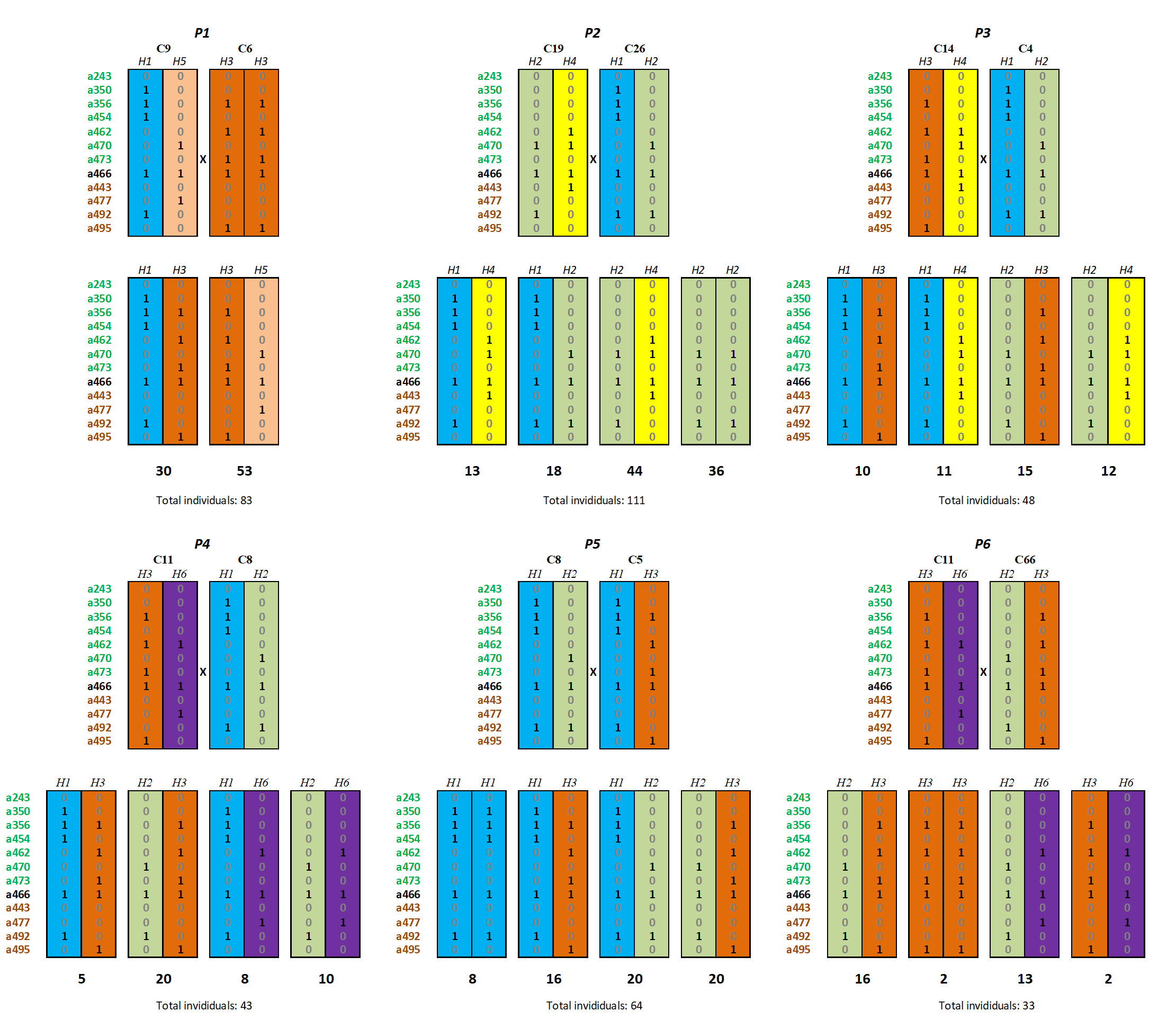

Supplement: Supplementary file 1 [file Data_Sheet_1.zip › Supplementary Figures/SF4. Observed segregating Haplotypes in six F1 populations.tif]

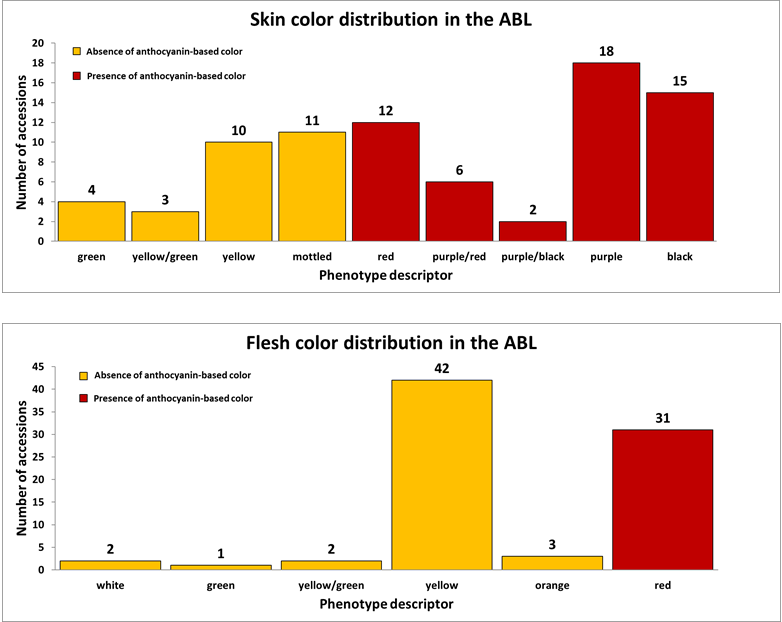

Supplement: Supplementary file 1 [file Data_Sheet_1.zip › Supplementary Figures/SF5. Phenotype distribution on the ABL.tif]

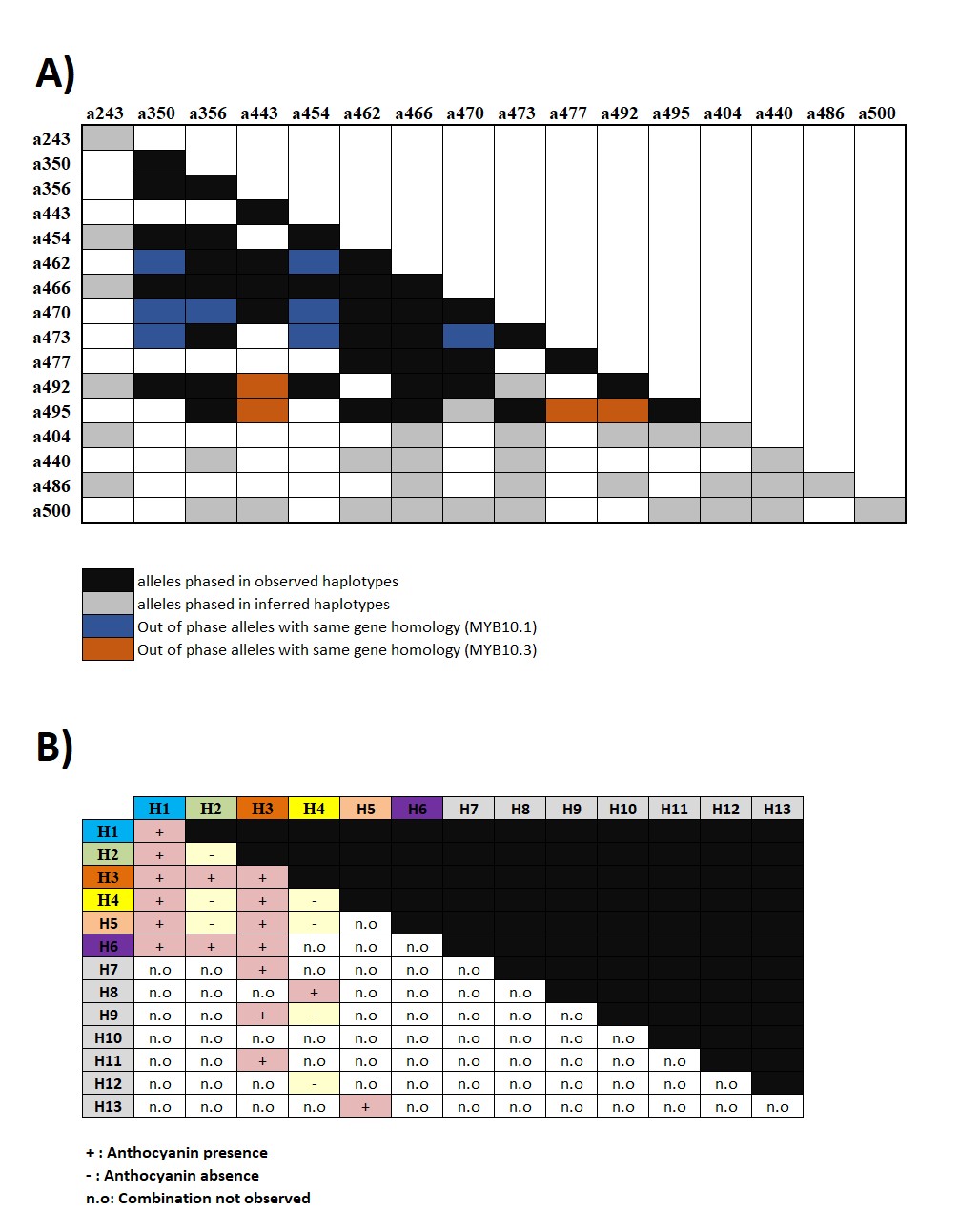

Supplement: Supplementary file 1 [file Data_Sheet_1.zip › Supplementary Figures/SF6. Allele (A) and Haplotype (B) combinations.jpg]
